# A meta-analysis of catalytic literature data reveals property-performance correlations for the OCM reaction

**DOI:** 10.1038/s41467-019-08325-8

**Published:** 2019-01-25

**Authors:** Roman Schmack, Alexandra Friedrich, Evgenii V. Kondratenko, Jörg Polte, Axel Werwatz, Ralph Kraehnert

**Affiliations:** 1 Technische Universität Berlin, Institut für Chemie, Str. des 17. Juni 124, 10623 Berlin, Germany; 2 Technische Universität Berlin, Institut für Volkswirtschaftslehre und Wirtschaftsrecht, FG Ökonometrie und Wirtschaftsstatistik, Straße des 17. Juni 135, 10623 Berlin, Germany; 3 Leibniz Institute for Catalysis (LIKAT Rostock), Albert-Einstein-Str. 29 a, 18059 Rostock, Germany; 4 Humboldt-Universität zu Berlin, Institut für Chemie, Brook-Taylor-Straße 2, 12489 Berlin, Germany

## Abstract

Decades of catalysis research have created vast amounts of experimental data. Within these data, new insights into property-performance correlations are hidden. However, the incomplete nature and undefined structure of the data has so far prevented comprehensive knowledge extraction. We propose a meta-analysis method that identifies correlations between a catalyst’s physico-chemical properties and its performance in a particular reaction. The method unites literature data with textbook knowledge and statistical tools. Starting from a researcher’s chemical intuition, a hypothesis is formulated and tested against the data for statistical significance. Iterative hypothesis refinement yields simple, robust and interpretable chemical models. The derived insights can guide new fundamental research and the discovery of improved catalysts. We demonstrate and validate the method for the oxidative coupling of methane (OCM). The final model indicates that only well-performing catalysts provide under reaction conditions two independent functionalities, i.e. a thermodynamically stable carbonate and a thermally stable oxide support.

## Abstract (web summary)

The incomplete nature and undefined structure of the existing catalysis research data has prevented comprehensive knowledge extraction. Here, the authors report a novel meta-analysis method that identifies correlations between a catalyst’s physico-chemical properties and its performance in a particular reaction.

## Introduction

Meta-analysis^1^ is a powerful tool to rigorously assess the findings of published research. Successful meta-analysis studies were reported in research fields where quantitative analysis of independently conducted experiments is prevalent, e.g., medical research^2^, genetics^3,4^, biology^5^, and economics^6^. Particularly in medical research, meta-analysis is used to aggregate individual studies aiming at the same treatment effect and employing the same research design (randomized control trials). In this case, the main goal and benefit of meta-analysis is to obtain a more precise and robust effect estimate than any individual study can deliver.

Despite the existence of a vast amount of well-documented experiments also in heterogeneous catalysis, hardly any corresponding meta-analyses have been reported. Efforts to collect and analyze sets of literature data have been reported primarily for the water gas shift reaction (WGS)^7^, CO-oxidation^8–10^, transesterification in biodiesel production^11^, and electro-catalytic oxidation of alcohols in direct alcohol fuel cells^12^. However, neither did these studies identify new structure–activity relationships nor could they provide simple chemical explanations for the observed statistical effects.

The shortcomings of these reports can be attributed, e.g., to (1) rather small datasets being used (data from 85 publications or less^7–12^), (2) statistical learning methods that search exploratively for relationships in the data, and (3) failure to incorporate existing chemical knowledge to inform and direct the statistical work. Another challenge faced in heterogeneous catalysis results (4) from the unsystematic heterogeneity of the available data. Each experimental report typically explores a narrow range of catalyst compositions and reaction conditions. However, the employed reaction conditions vary widely between different publications, which influences the catalytic performance via chemical kinetics, and makes a meta-analysis challenging.

A significant break-through in terms of the dataset quality was reported by Zavyalova et al. in 2011^13^ and in a recent follow up^14^ (see Supplementary Notes 1 and 2 for details). The dataset of Zavyalova et al.^15^ was made freely available and covered 1866 distinct catalyst compositions collected from 421 reports. The dataset provides for the oxidative coupling of methane (OCM) information on catalyst composition, reaction conditions and catalyst performance. The study employed formal mathematical approaches (multiway ANOVA, Pearson’s and Spearman’s correlation coefficients, regression trees) to search for correlations between catalyst composition and catalyst performance. The study concluded that some combinations of key elements contained in a catalyst can contribute to high C_2_ selectivity or yield, but did not result in a general model that correlates catalyst composition to catalytic performance with proven statistical significance and robustness. Unfortunately, a simple correlation between elemental composition and performance is also not likely to exist, since materials with the same nominal composition can often adopt very different structures and surface terminations that should result in different OCM performances. Hence, descriptors other than just elemental composition are needed.

We report a meta-analysis method that can identify statistically significant correlations between the physico-chemical properties assigned to a catalyst material and its performance in a given reaction. The method employs three distinct sources of information to achieve this goal. It incorporates in addition to (i) the experimental data reported in literature also (ii) general textbook knowledge about fundamental material properties and (iii) the experienced intuition of a chemist or material scientist about possible property–performance correlations. The OCM is used here as an example to illustrate the approach and derive property–performance correlations.

## Results

### Meta-analysis approach

**Fig. 1:**
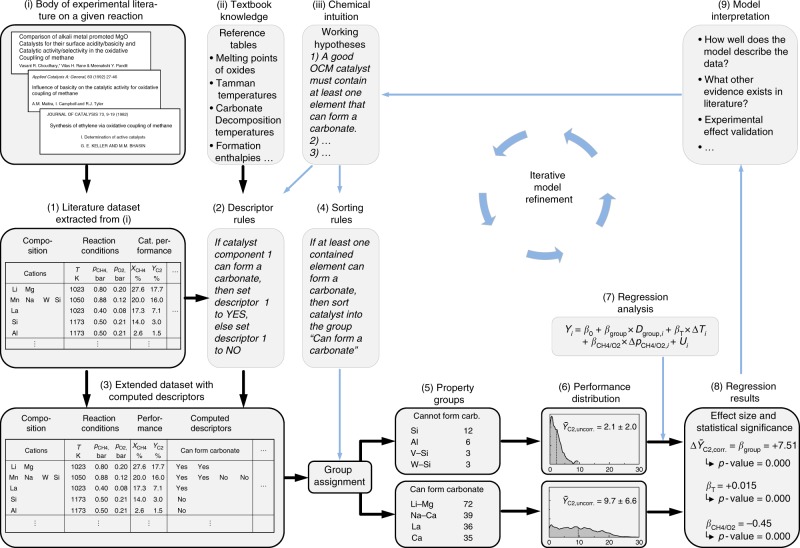
Proposed meta-analysis approach. Starting out with (i) experimental catalytic data reported in literature, (ii) general textbook knowledge and (iii) a researchers chemical intuition a working hypothesis is formulated, processed, tested against the data for its statistical significance, and iteratively refined into a property–performance model (steps 1–9). Bold arrows mark the flow of the data. All specific examples relate to the studied OCM reaction and hypothesis (1). (see Method section and Supplementary Notes 5–9 for further details)

The method is schematically outlined in Fig. 1. It starts out from the chemist’s intuition expressed as a hypothesis (iii) about a supposed relationship between the properties of a catalyst material and its catalytic performance. In a first step, data on the composition, reaction conditions and performance of different catalysts are assembled from the body of literature (i) into a dataset (1) for this reaction. Then, related textbook knowledge (ii) on all relevant elements is collected in the form of tables that provide, e.g., the ability of an element to form certain types of chemical compounds (e.g., oxides, carbonates), their thermal stability, formation enthalpies, and melting points. Moreover, descriptor rules (2) are derived that define how to calculate the so-called physico-chemical descriptors that are required to express the hypothesis (iii). Applying the descriptor rules to each catalyst entry in the dataset creates an extended dataset (3) that includes for each catalyst additional physico-chemical property descriptors. One particular strength of this procedure is the fact that the descriptors can be computed as a function of temperature and pressure, i.e., at conditions that closely reflect the individual conditions at which a catalyst was experimentally tested in the original experimental report. Hence, the computed properties closely reflect the state of the catalyst during the actual performance testing.

The chemical hypothesis (iii) can now be translated into a set of formal sorting rules (4). These sorting rules express in a formal way, which descriptors are assumed to correlate with high catalytic performance. More precisely, a sorting rule states that a catalyst will be assigned to one specific property group (5) if the catalyst’s descriptors possess values within a defined range. Applying the rules to the extended dataset (3) divides the data into smaller subsets of so-called property groups (5). Then, the measured performance value of each catalyst is used to compute for each property group a density distribution of the performance indicator (6), its averaged value as well as a standard deviation. If the descriptors chosen in step 4 represent properties that are indeed relevant to the catalysts performance, then the performance distributions (6) should differ significantly between the corresponding property groups. However, these performance distributions do not account yet for the fact that each catalyst was measured at different reaction conditions.

In order to compensate for these differences in reaction conditions, a multivariate regression analysis (7) is invoked that approximates the influence of, e.g., temperature (*T*) and the ratio of feed gases (here: *p*_CH4_/*p*_O2_). Moreover, it considers to which of two compared property groups a given catalyst had been assigned. The regression procedure adjusts all regression coefficients *β*_*i*_ in order to minimize the deviation between experimental and computed yield values over all entries in the two groups. The computed regression results (8) quantify the influence of temperature (*β*_*T*_), pressure ratio (*β*_CH4/O2_), and most importantly, the effect of belonging to either one of the two compared catalyst groups (*β*_group_) on the catalysts performance (*Y*_C2_). The results significance is judged via a *t*-test that calculates for each *β*-regression coefficient a corresponding *p*-value. Low *p*-values (*p* < 0.05) indicate a high statistical significance, with, e.g., *p* < 0.05 implying a confidence exceeding 95% that two compared catalyst groups are indeed distinctly different in their performance (All *p*-values reported in this paper were obtained via *t*-test, see method section and Supplementary Note 8 for further details.).

In step (9), the obtained models are compared to additional spectroscopic, computational and fundamental evidence from literature. An iterative refinement of hypotheses (iii), descriptors (3), sorting rules (4) and if possible also the studied data (2) results in robust and statistically significant property–performance models.

### Literature OCM dataset

The oxidative coupling of methane aims to convert methane and oxygen selectively into C_2_ coupling products (ethane, ethylene) while avoiding total oxidation^16^. The OCM reaction is catalyzed by numerous materials with very diverse elemental compositions^17^. Economic viability of the process would require C_2_ yields well above the best values (ca. 30%) reported so far^17,18^. Despite decades of research, the reaction mechanism is not fully understood even for extensively studied catalyst such as Li/MgO and Mn-Na_2_WO_4_/SiO_2_^19,20^. It is generally presumed that the reaction network involves gas-phase reactions coupled to reactions at the catalyst surface^21–23^, where surface defects facilitate C–H bond activation. Unfortunately, the required high reaction temperatures (500 to 1000 °C)^20^ obstruct many common in situ analytical tools. Hence, definite proof exists so far neither for the relevance of such defects under practical operation conditions nor the critical contribution of gas-phase reactions. Moreover, no generally valid correlation between a catalyst’s composition, its structure and its OCM performance has been established yet.

**Fig. 2:**
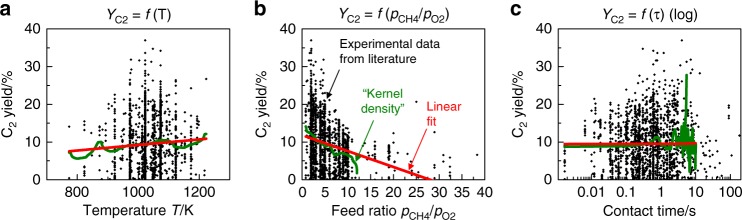
Distribution of data in the corrected OCM dataset (1802 data points). The data are plotted in terms of C_2_ yield **a** as a function of temperature (*T*), **b** pressure ratio methane/oxygen in the feed gas (*p*_CH4_/*p*_O2_) and **c** contact time (τ, log). Each point represents one individual catalyst. Red lines indicate a linear fit. Green lines represent a so-called Epanechnikov kernel density function

We illustrate the developed meta-analysis approach in a complete re-analysis of the OCM data compiled by Zavyalova et al ^13^ after corrections and outlier removal from the data (see Supplementary Note 3). The data is represented by a table that contains 1802 rows (different catalyst compositions) and 37 columns (see Supplementary Note 4 and Supplementary Data 1). The columns encode for each catalyst (a) the elements contained in the catalyst and their molar fractions, (b) parameters indicating the reaction conditions (*T*, *p*_CH4_, *p*_O2_, *p*_CH4_/*p*_O2_, *p*_total_, contact time) and (c) numerical values that describe catalyst performance (*X*_O2_, *X*_CH4_, *S*_CO*x*_, *S*_ethane_, *S*_ethene_, *S*_C2_, *Y*_C2_). Out of these categories, elemental composition, reaction temperature (*T*), reactant partial pressure  ratio (*p*_CH4_/*p*_O2_) and C_2_ yield (*Y*_C2_) were evaluated. Figure 2 displays for the corrected dataset exemplarily *Y*_C2_ as a function of (a) reaction temperature, (b) *p*_CH4_/*p*_O2_, and (c) contact time to illustrate the covered data range and the data heterogeneity. (See also Supplementary Note 20 for individual catalyst examples Li–Mg and Mn–Na–W–Si.)

### Derived final model

**Fig. 3:**
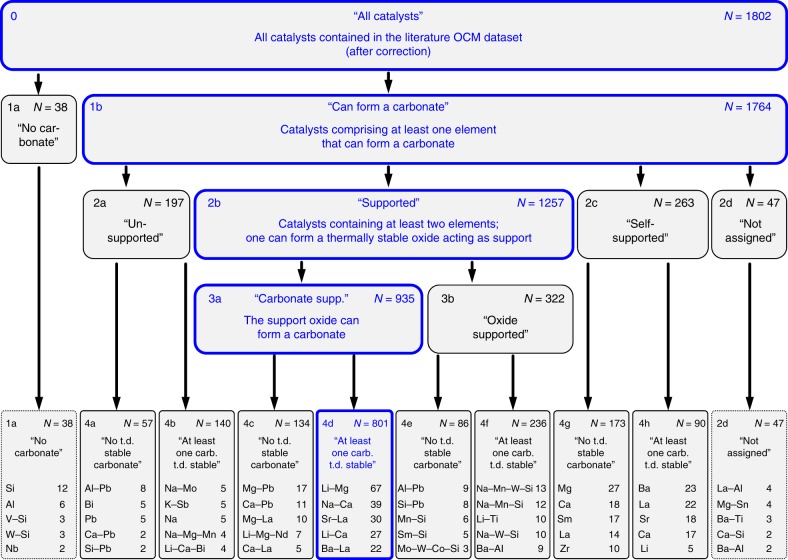
Tree representation of the best derived OCM property–performance model. Each box represents a distinct property subgroup obtained from the applied sorting criteria. Starting out from the initial dataset (group 0), each catalyst is assigned to one out of ten terminal subgroups (1a; 4a–4h; 2d). For each terminal subgroup also the five most frequent catalyst compositions (i.e., cation combinations) and the corresponding number of observations are listed. *N* denotes the number of catalysts in each group. Blue colored bold frames mark the group with the highest averaged C_2_ yield on each hypothesis level. “t.d.” and “carb.” refer to “thermodynamically” and “carbonate”, respectively. A full list of catalyst compositions (i.e., cation combinations) is provided in Supplementary Data 5

Numerous hypotheses were tested. The derived best model employs four different hypotheses to divide the OCM dataset into a total of 18 different property groups as illustrated by the tree-like representation shown in Fig. 3. Each displayed box represents a group of *N* catalysts. Starting out from the complete dataset (group 0, *n* = 1802), the physico-chemical criteria related to hypothesis (1) divide the contained catalysts into two subgroups (groups 1a, 1b). The subsequently applied hypotheses (2), (3), and (4) divide these subgroups further, resulting in 10 terminal subgroups 1a, 4a…4h, and 2d. For each terminal subgroup, the respective five most frequent catalyst compositions are listed in Fig. 3 (see Supplementary Note 10 for a complete list of the catalysts contained in each group). Bold box frames indicate on each level the catalyst group that shows the best catalytic performance in terms of average C_2_ yield.

**Fig. 4:**
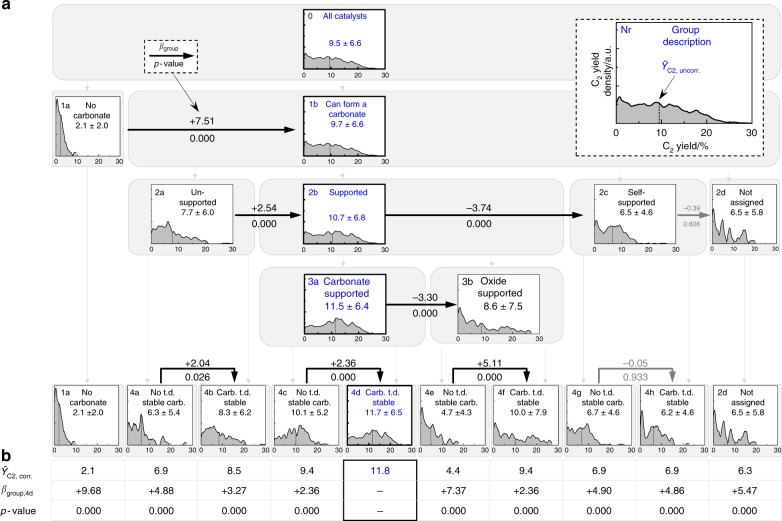
Statistical evaluation for the best derived property–performance model. **a** Tree representation of the model with the same property groups as shown in Fig. 3. Diagrams display for each property group the C_2_ yield–density distribution, the mean C_2_ yield *Ȳ*_C2,uncorr_, and its standard deviation (marked by ±). The arrow labels provide exemplarily the regression results, i.e., the value of *β*_group_ derived from the regression analysis, which corresponds to a difference in mean yield (~ Δ*Ȳ*_C2, corr_) between the two groups after correction for the influence of temperature *T* and pressure ratio *p*_CH4_/*p*_O2_. Moreover, the *p*-value corresponding to *β*_group_ is displayed, where smaller *p*-values represent a higher statistical significance. Blue color marks on each hypothesis level the group with the highest mean C_2_ yield. “t.d.” and “carb.” refer to “thermodynamically” and “carbonate”, respectively. **b** Final model outcome in terms of performance (*Ȳ*_C2,corr_) for each terminal group. Regression coefficient *β*_group_ and *p*-values are reported for the comparison of the best-performing group 4d with each other terminal subgroup. Note that for reasons of clarity only a fraction of the performed tests is shown. The complete model contains each possible pairwise comparison among all formed subgroups. The complete list of all obtained regression coefficients (*β*_group_, *β*_*T*_, *β*_CH4/O2_, and the respective *p*-values are provided in Supplementary Note 11a/b/c)

Figure 4a displays in a similar representation the most important results of the regression analysis and statistical evaluation that produced the final model displayed in Fig. 3. For each group, a diagram is provided that illustrates the C_2_ yield distribution within the group along with the mean C_2_ yield (${\bar{{Y}}}$_C2,uncorr_, marked by a dashed line, i.e., *Y*_C2_ prior to regression analysis) and its standard deviation. The horizontal arrows that connect two respective boxes represent the performed statistical tests. Figure 4b summarizes the final model outcome in terms of performance (${\bar{{Y}}}$_C2,corr_) for each terminal group. All observed *p*-values are lower than 0.005, which underlines the extremely high statistical significance of the derived final model.

*Hypothesis 1—ability to form at least one carbonate compound*: An initial assessment of the dataset gave the impression that many OCM catalysts contain components that can form a carbonate. Hypothesis 1 therefore proposes that a good OCM catalyst contains at least one chemical element that is known to form a carbonate compound.

All catalysts that can form at least one carbonate were assigned to group 1b “Can form a carbonate”, all other catalysts to group 1a “Cannot form a carbonate” (Fig. 3). Only a small fraction of catalysts (*n* = 38) is not able to form any carbonate, whereas the vast majority of reported catalysts can form at least one carbonate (1b: *n* = 1764) (Fig. 3).

Catalysts that can form at least one carbonate (1b) show on average a 7.6 % higher yield of C_2_ hydrocarbons (${\bar{{Y}}}$_C2,uncorr_ = 9.7 %) than catalysts in group 1a (${\bar{{Y}}}$_C2,uncorr_ = 2.1 %). The difference in C_2_ yield computed via multiple regression analysis (Δ${\bar{{Y}}}$_C2,corr_ = *β*_group_ = + 7.51) proves to be statistically highly significant on a >99.5 % confidence level (*p* < 0.005). Also the regression coefficients obtained for temperature (*β*_*T*_ = +0.0015; *p* = 0.000) and pressure ratio *p*_CH4_/*p*_O2_ (*β*_CH4/O2_ = −0.452; *p* = 0.000) show high statistical significance with higher C_2_ yields corresponding to higher temperatures and higher oxygen partial pressures (see Supplementary Note 9 for a discussion and interpretation).

Group 1a contains mostly Si, Al, V, W, Nb, Sn, Pt, Ti, and combinations thereof (Fig. 3), i.e., elements typically not associated with high OCM activity. In contrast, group 1b includes all the usual suspects of high OCM performance, i.e., the Li/MgO system (Li–Mg), catalysts based on lanthanum oxide and calcium oxide, as well as variations of the Mn–Na_2_WO_4_/SiO_2_ system (see Supplementary Note 10 for full list). Hence, hypothesis 1 is able to sort catalysts that are not very active (silica, alumina) or combust methane unselectively (vanadium on silica, Pt) into group 1a using an objective chemical criterion. Thus, statistical noise is removed from the remaining set of catalysts (1b).

*Hypothesis 2—presence of a stable oxide acting as a support*: Many reported OCM catalysts feature two components. One of the components often appears to be able to form a thermally stable metal oxide. Such metal oxides are frequently used in heterogeneous catalyst to support an active phase. Hypothesis 2 therefore defines two criteria that attempt to describe a property “catalyst support”. Criterion (i) states that among all the oxides that can possibly be formed by a catalyst’s cations, at least one oxide is present that possesses a Tammann temperature higher than the individual temperature at which the catalyst was tested in OCM. An additional criterion (ii) imposes that the mass content of this stable oxide amounts to at least 50 wt% of the catalyst’s constituents, assuming that they are present in the form of their respective most stable oxides. An additional criterion (iii) used for the group assignment is the presence of an element other than the designated support oxide, and its ability to form a carbonate.

A catalyst was assigned to group 2a “unsupported” (*n* = 197) if none of a catalyst’s components fulfilled both support criteria (i) and (ii) (Fig. 3). Group 2b “supported” (*n* = 1257) represents all catalysts where one component fulfils both support criteria (i, ii), and where an additional cation is present that is able to form a carbonate. Catalysts were assigned to group 2c “self supported” (*n* = 263) when a catalyst contained only one component, and if this component satisfied both support criteria AND could form a carbonate. All remaining catalysts were assigned to group 2d “not assigned” (*n* = 47) for further noise reduction.

Catalysts in group 2b “supported” showed higher C_2_ yields (${\bar{{Y}}}$_C2,uncorr_ = 10.7%) than catalysts in the group 2a “unsupported” (${\bar{{Y}}}$_C2,uncorr_ = 7.7%) and group 2c “self-supported” (${\bar{{Y}}}$_C2,uncorr_ = 6.5%). The differences are statistically highly significant.

The best-performing group (2b “supported”) features the highest number of catalyst entries (*n* = 1257) and contains most of the classical OCM catalysts (e.g., Li/MgO, Mn–Na_2_WO_4_/SiO_2_). Most group entries consist of a binary combination of La or alkali metals with an alkaline earth element (Li–Mg, Na–Ca, Li–Ca). Typically, the support criteria are satisfied by the thermally stable oxide of the alkaline earth element. Many of the cation combinations that include heavy metals (Pb, Bi) and alkali elements (Li, Na, K, Cs) are assigned to group 2a “unsupported” because their respective (super)oxides fail the thermal stability criterion (i), or because the stable oxide is not present in sufficiently large amounts (ii). The “self-supported” catalysts 2c contain only a single cation, which is either a lanthanide or an alkaline earth element, and forms a thermally stable oxide. The 47 catalysts placed in group 2d “not assigned” comprise mostly binary combinations that contain a large amount of a stable support oxide, but fail criterion (iii) because the second element (that is not the designated support) cannot form a carbonate.

*Hypothesis 3—ability of the support oxide to form a carbonate*: Hypotheses 1 and 2 suggest that the ability to form a carbonate plays a critical role in OCM catalysis. All catalysts retained in group 2b contain at least one element that can form a carbonate, and another element that can form a thermally stable oxide. Hypothesis 3 tests, if also for this support oxide the ability to form a carbonate impacts a catalyst’s OCM performance.

All catalysts for which the designated support oxide can form a carbonate were assigned to group 3a “carbonate-supported” (*n* = 935), all other catalysts to group 3b “oxide-supported” (*n* = 322). Most of the “carbonate supported” catalysts consist of La or an alkali element combined with an alkaline earth element where typically the alkaline earth elements provide the support functionality. The “oxide-supported” catalysts (the designated support oxide cannot form a carbonate) comprise mostly alkali and alkaline earth elements or Na_2_WO_4_ supported on either alumina, silica or titania (see element combinations listed in groups 4e and 4f in Fig. 3).

The carbonate-supported catalysts (group 3a, ${\bar{{Y}}}$_C2,uncorr_ = 11.5%) clearly outperform the oxide-supported group 3b (8.6%) by about 2.9% C_2_ yield (*β*_group_ = -3.30) and with high statistical significance (*p* = 0.000), providing further evidence for the beneficial and important role of carbonates in OCM catalysis.

*Hypothesis 4—thermodynamic stability of carbonates during OCM*: A property often attributed to good OCM catalysts is their so-called basicity^24–27^. One possible measure for the basicity of a metal oxide is its ability to form a carbonate and the carbonate’s thermal stability. If such a carbonate is thermodynamically stable also at the high temperatures of OCM, then the carbonates are likely to be present on the catalyst surface during the OCM reaction.

Hypothesis 4 therefore assesses the thermodynamic stability of all carbonates that can be formed by the elements present in a catalyst by comparing the decomposition temperature of the most stable carbonate to the temperature at which the OCM performance was measured. A catalyst is assigned to a subgroup “carbonate thermodynamically stable” if the OCM test was performed at a reaction temperature that is lower than the temperature at which the most stable contained carbonate decomposes (corrected for a constant offset that accounts for experimental uncertainties and dependence of carbonate decomposition equilibria on CO_2_ partial pressure, here: *T*_OCM measurement_ < *T*_carbonate decomposition_ + 100 K; for other offsets see Supplementary Note 14h-k).

Hypothesis 4 was applied to all subgroups that had been derived via positive sorting criteria in the previous hypotheses (i.e., not to the “not-groups” 1a and 2d). It divides the groups 2a, 3a, 3b, and 2c into the respective subgroups 4a, c, e, g (“no thermodynamically stable carbonate”), and subgroups 4b, d, f, h (“at least one thermodynamically stable carbonate”) (Fig. 3). Three out of four corresponding comparisons, i.e., 4a/4b, 4c/4d, 4e/4f, show a strong statistical significance indicated by *p*-values being equal or lower than 0.026 (Fig. 4a). In all three statistically significant cases the catalysts that can form at least one thermodynamically (“t.d.”) stable carbonate outperform the corresponding catalysts that cannot form a stable carbonate, with C_2_ yields ${\bar{{Y}}}$_C2,corr_ being higher by 2.04% (4a/4b), 2.36% (4c/4d), and 5.11% (4e/4f) (see *β*_group_ values in Fig. 4a).

Despite the fact that the two most common OCM catalyst types (Li/MgO, Mn–Na_2_WO_4_/SiO_2_) were assigned to different groups on level 3 (Li–Mg: 3a; Na–Mn–W–Si: 3b), both end up in the respective subgroups that can form at least one t.d. stable carbonate (Li–Mg: 4d; Na–Mn–W–Si: 4f). In general, the role of the thermodynamically most stable carbonate is typically fulfilled by alkaline, alkaline earth, or lanthanide elements in the subgroups 4b/d/f/h. In absence of these elements no thermodynamically stable carbonate can be formed and lower yields are observed (see Supplementary Note 13 for a more detailed discussion).

Hypothesis 4 thus illustrates another strength of the method: the same sorting criteria can be applied to different groups, in this case groups derived on hypothesis levels 2 and 3. Consistent results obtained across different groups point towards a general validity of the proposed effect.

### Model robustness

The robustness of the final model (Figs. 3 and 4) was assessed using different approaches of parameter variation, changing the independent variable to ln(*Y*_C2_) and with a so-called “robust regression” (see Supplementary Notes 14–18 for details). The model retained the same statistically significant correlations, except for few instances where some correlations on level 4 lost their statistical significance. The final model is therefore highly robust despite the data heterogeneity, potential publication bias within the data and the small size of some of the formed terminal groups.

## Discussion

The derived model reveals clear property–performance correlations. It establishes for the first time a generalized set of physico-chemical properties that discriminate between high- and low-performing OCM catalysts. Four simple hypotheses suffice to divide 1802 catalysts into 10 groups of distinct properties and OCM performance (Fig. 4b). Following the path of the best-performing groups on each hypothesis level indicates a combination of properties that correlates with high C_2_ yields. The model predicts the highest C_2_ yield (4d, ${\bar{{Y}}}$_C2, corr_ = 11.8%) when (1b) at least one of the elements contained in the catalyst can form a carbonate, (2b) the catalyst contains an additional element that forms a thermally stable oxide, (3a) this oxide is also able to form a carbonate, and (4d) at least one of the possible carbonates is thermodynamically stable at the applied OCM reaction temperature. Also a comparison of the best-performing terminal group 4d with each other terminal group (1a, 4a-h, 2d) in terms of yield difference (*β*_group_) and *p*-value shows a very high statistical significance (*p* < 0.005).

The nature of the hypothesis formulation in terms of chemical conditions facilitates a direct physico-chemical interpretation. Good OCM catalysts comprise at least two elements, with one of the elements being able to form a carbonate at the temperatures of OCM reaction, and a second element that can form a thermally stable (non-sintering) oxide under OCM conditions (hypothesis 2). Good OCM catalysts thus provide two functionalities, i.e., a “support” and an “active phase”. The thermally stable oxide (alkaline earth and lanthanide oxides, alumina, silica, titania) acts as support and provides a high surface area during OCM (2b). The active phase appears to be related to the ability to form a carbonate. The ability to from a carbonate^24^ is one out of several possible descriptors for basicity (Smith scale^28^; optical basicity^29^, an ionic-covalent parameter^30^). Basicity has been proposed by many authors as an important feature of OCM catalysts^13,24–26,31^. However, no general quantitative model for the impact of basicity on OCM performance has been established so far.

The ability to retain a stable carbonate at the studied OCM temperature results in higher C_2_ yields (hypothesis 4). Hence, the actual presence of carbonates during OCM is likely. Carbonates could therefore contribute to the reaction, i.e., via ongoing transitions from carbonate into oxide and vice versa, which would continuously produce new defect sites^32^. However, the presence of carbonate phases can assist also the formation and stabilization of peroxide species^33–36^. Such peroxides species could activate methane in a selective fashion to generate methyl radicals^32,35,37^. Otsuka et al.^38,39^ reacted CH_4_ with bulk peroxides (Na, Sr, Ba) and observed C_2_ coupling products already at 400 °C.

C_2_ yields benefit also from the ability of the support oxide to form a carbonate (3a “carbonate-supported”). Possibly, carbonate formation suppresses unselective combustion of methane and C_2_ hydrocarbons, which was reported to occur on oxide-supports (Al_2_O_3_^40^, SiO_2_^41^, and TiO_2_^42^, group 3b “oxide-supported”).

The essential role of carbonates in the model provides a clear incentive to rethink the classical OCM reaction mechanism and in particular the role of CO_2_. Carbon dioxide was so far typically regarded only as an undesired byproduct. However, the only way that carbonates can form during OCM is a reaction with CO_2_. Hence, CO_2_ could be essentially required to stabilize carbonate phases that contribute directly to C_2_ formation or prevent further oxidation of C_2_ products. This could provide also a new explanation of the observation that a maximum of ca. 30% C_2_ yield has not been overcome so far when feeding CH_4_/O_2_ mixtures to the reactor^17^. Methane combustion would be essentially required to form CO_2_ and the carbonate phase, thus limiting the achievable C_2_ yield. Supplying the required CO_2_ to the catalyst via CO_2_ co-feed instead of methane combustion could provide a simple path to improved C_2_ yields.

**Fig. 5:**
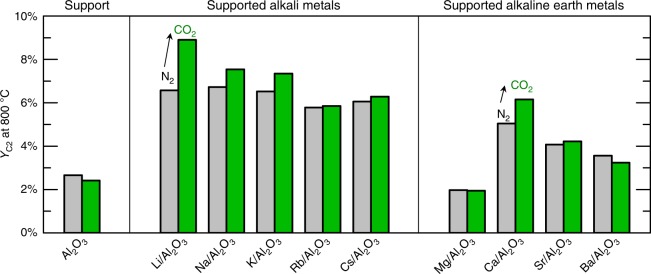
Experimental verification of the influence of CO_2_ co-feed in OCM. The effect of adding CO_2_ to the OCM feed gas was measured for nine different alumina-supported catalysts as well as the bare alumina support. The yield of C_2_ hydrocarbons (ethane plus ethylene) measured at 800 °C is plotted for two different feed conditions, i.e. with CO_2_ co-feed (green bars, CH_4_/O_2_/N_2_/CO_2_ = 26.2: 14.8: 3.0: 56.0 and without CO_2_ co-feed (gray bars, CH_4_/O_2_/N_2_ = 26.2: 14.8: 59.0). Within the limits of experimental accuracy, CO_2_ addition either increases *Y*_C2_, or shows a negligible effect for all studied catalyst. All catalysts were prepared and measured under identical conditions, changing only the precursors employed to support the different active metals

The influence of CO_2_ was experimentally verified. The stability of a carbonate depends on both temperature and CO_2_ partial pressure. Increasing the CO_2_ partial pressure should increase the carbonate’s stability, which, according to the model, should increase the C_2_ yield. We tested this effect experimentally using nine different catalysts belonging to group 3b (see Supplementary Note 19). Figure 5 compares the C_2_ yields measured at 800 °C, both in the absence and in the presence of CO_2_ co-feed. When carbon dioxide is added to the OCM feed the observed C_2_ yields either increase (by up to 2.5%) or remain constant. For the alumina support alone, which cannot form a carbonate, CO_2_ addition does not have a measurable impact. The experimental data further support the model interpretation that carbonates are likely to be formed during OCM and result in higher C_2_ yields.

The following paragraphs discuss the power of the developed meta-analysis method as well as current limitations. The exemplarily performed evaluation of 1802 observations in OCM catalysis provides a clear picture of physico-chemical properties that have a statistically significant impact on the observed C_2_ yield. What sets the presented meta-analysis apart from previous work is that chemical knowledge and chemical reasoning become an integral part of the analysis, i.e., a chemical instead of mathematical perspective.

The method yields a robust and meaningful model, with C_2_ yield distributions becoming narrower with each model level. Yet, a considerable variety of C_2_ yields remains within each property group. This could be improved by further refined hypotheses, additional property descriptors, or more complicated combinations/interactions of the catalyst constituents.

A substantial contribution to the broadness of the distribution results from the variance in the underlying data (see Supplementary Note 20 for yields reported, e.g., Li/Mg and Mn/Na/W/Si). This could result from the diversity of employed experimental conditions, but also subtle changes in the synthesis procedure that can strongly influence the structure and performance of a catalyst. In future work it would be desirable to incorporate also intricate details such as catalyst structures, synthesis recipes or kinetic information.

One major advantage of our approach is the combination of experimental data with sound chemical textbook knowledge. Thus, hypotheses with a high level of abstraction can be formulated. Pioneering work of, e.g., Schueth^43–46^, Rothenberg^47–51^, and Baumes^52,53^ provides access to more advanced descriptors. Also descriptors based on DFT calculations^54–58^, e.g., efficiently computed d-band centres^59^, could be incorporated. Alternative basicity descriptors such as the Smith scale^28^, optical basicity^29^, or the ionic-covalent parameter^30^ should be implemented to test whether OCM performance is primarily governed by basicity or indeed the presence of carbonates. In the future, hypotheses could be generated via rapidly improving algorithms and machine-learning approaches (see e.g., refs. ^60–62^ for excellent reviews), or following schemes suggested by Rothenberg and co-workers^50,63,64^.

In general, statistical tools can explore only correlations between variables that are quantitatively accessible. Quantity, quality, and distribution of literature data could be improved by adopting standardized testing and benchmarking procedures for a particular reaction^65,66^, depositing complete sets of original data in open repositories and using data formats designed for facile data sharing^67^.

The developed meta-analysis method allows the identification of catalytic property–performance correlations hidden in the vast body of existing experimental research. Applying the method to the OCM, we identify general correlations between a material’s physico-chemical properties and its OCM performance. Good catalysts comprise at least two elements, with one element being able to form a thermodynamically stable carbonate at the temperatures of OCM reaction, and a second element forming a thermally stable (non-sintering) oxide under OCM conditions. Good catalysts apparently require a support that provides a high surface area at OCM temperatures, and carbonate(s) that either contribute directly to C_2_ formation and/or prevent subsequent unselective oxidation of the C_2_ products. Future work could assess the specific role of CO_2_ and carbonates in OCM using, e.g., in-situ spectroscopic experiments or quantum-chemical calculations. In a broader context, the developed meta-analysis method provides a generic and open platform for data science in heterogeneous catalysis. The concept allows the extensions to other reactions, more complex and fundamental descriptors derived, e.g., from quantum chemistry, and ultimately also the incorporation of a catalyst’s structure. Consequently, it could empower all chemists to work as data scientists and explore the full potential of their valuable experimental data.

## Methods

### Dataset corrections

The dataset compiled by Zavyalova et al.^13^ was corrected for errors (wrong temperature or composition, duplicate entries) based on a comparison with the original literature. Some entries were removed that contained highly unusual feed compositions and contact times, lacked essential information (*T*, *p*_CH4_, *p*_O2_) or were not measured in continuous-flow with methane and oxygen co-feed. Moreover, we removed the predefined data categories “promotor” and “support”, to which Zavyalova et al.^13^ had assigned some elements, and reassigned the respective elements to the more general categories “cation” and “anion” (see Supplementary Note 3 and Supplementary Note 4). The corrected dataset is available as Supplementary Data 1 (20160720_corrected-dataset_.xls).

### Physico-chemical properties

The physico-chemical properties of individual chemical elements and chemical compounds formed by these elements were compiled from literature and own measurements. The properties include the molar mass of an element, its position in the periodic table, the ability to form a carbonate, and the carbonate’s decomposition temperature (thermal stability, indicator for basicity). Moreover, properties of potentially formed oxides were incorporated (oxidation number, the stoichiometry of the most stable oxide, and the oxide’s melting point). From this melting point, the Tammann temperature^68^ was computed as a measure of the onset of sintering and loss of surface area. Supplementary Note 5 describes the complete property table, the respective data are provided as Supplementary Data 2 (20160811_element-properties.xls).

### Physico-chemical descriptors

The physico-chemical descriptors were computed by a set of instructions that combine values extracted from the OCM catalyst table as well as the elemental-property table. The descriptor values were written into new columns (so-called dummy variables) of an extended data file. Important descriptor categories employed in the present study are the ability of each catalyst component (e.g., “cation”) (i) to form a carbonate compound, (ii) the thermal stability of this carbonate, and (iii) the ability to form a sufficiently stable oxide that could act as catalyst support. A value “true” (“1”) is assigned to a carbonate descriptor (i) if the respective catalyst component is able to form a carbonate. Descriptor (ii) describes whether this carbonate is stable under OCM reaction conditions. A value “true” is assigned if the OCM reaction is measured at a temperature that is lower than the actual temperature at which the carbonate starts to decompose, corrected for an offset that accounts for experimental uncertainties (*T*_OCM measurement_ < *T*_carbonate decomposition_ + 100 K). A “true” value is assigned to the respective support descriptors (iii) if the catalyst contains a cation that can form a thermally stable oxide, i.e., an oxide that possesses a Tammann temperature (*T*_Tammann_ = *T*_melt,oxide_ × 0.6) higher than the actual temperature at which the OCM data were measured for the catalyst, and if the concentration of this oxide amounted to at least 50 wt% of the catalysts constituents, each in the form of its most stable oxide. The descriptor rules take the reaction temperature explicitly into account for each catalyst, hence the same descriptor can adopt different values depending on the actual conditions under which a catalyst was measured. Supplementary Note 6 provides further details on descriptor computation and resulting values. Respective data are provided in Supplementary Data 3 (20160729b_corrected_preprocessed_dataset_descriptors_.xlsx).

### Sorting rules

Sorting rules assigned each catalyst entry to one of at least two distinct “property groups” based on the values of the corresponding descriptor variables. In the studied OCM example, sorting rules were implemented to generate property groups that, e.g., (1) contain at least one element that can form a carbonate (“can form carbonate”), that (2) contain at least one additional element that fulfils the proposed support criterion of a stable and abundant oxide (“supported”), evaluate (3) whether the element that has been assigned the support function is also able to form a carbonate (“carbonate-supported”), and probe (4) whether the carbonate with the highest decomposition temperature is thermodynamically stable under the respective OCM conditions. Thus, very complex and hierarchically nested hypotheses can be formulated. Supplementary Note 7 discusses the employed sorting rules in detail. Respective data are provided in Supplementary Data 4 (20160729b_corrected_preprocessed_dataset_property_groups.xlsx).

### Regression analysis

A multiple regression analysis was employed in order to quantify the performance difference between catalysts assigned to two different property groups while compensating also for the effect of differences in the OCM measurement conditions. OCM catalysts are often compared based on the obtained yield of C_2_ products. However, it is obvious from chemical kinetics that the measured C_2_ yield is critically influenced not only by a catalyst’s composition, but also the reaction conditions (*T*, *p*_CH4_, *p*_O2_, *p*_total_, contact time) employed in a catalytic test. Due to the lack of standardized OCM test protocols, employed operation conditions vary significantly between different literature reports. Unfortunately, the limited amount of catalytic data available in the studied dataset did not allow a complete kinetic description for each catalyst. In order to mitigate this problem to some extent, the regression equation (1) implements a linear correction for the variables “ratio *p*_CH4_/*p*_O2_” and “temperature”:

1 $$Y_i = \beta _0 + \beta _{{\mathrm{group}}} \times D_{{\mathrm{group}},i} + \beta _T \times \Delta T_i + \beta _{{\mathrm{CH}}4/{\mathrm{O}}2} \times \Delta p_{{\mathrm{CH}}4/{\mathrm{O}}2,i} + U_i$$

whereas the dummy variable *D*_group,*i*_{0, 1} describes to which of the two compared property groups each catalyst *i* had been assigned. The regression procedure then adjusts all *β* values in order to minimize the sum of squares of the residuals *U*_*i*_ between experimental (*Y*_*i*_) and computed yield values over all entries in the two groups. The derived *β* values quantify the influence of temperature (*β*_*T*_), pressure ratio (*β*_CH4/O2_), and most importantly, the effect of belonging to either one of the two compared catalyst groups (*β*_group_) on *Y*_C2_. Hence, *β*_group_ is the obtained quantitative measure (in terms of corrected C_2_ yield) for the influence of a set of physico-chemical properties on the catalytic performance in OCM.

### Significance testing

The statistical significance of information derived from each *β* regression coefficient was judged via a *t*-test. The *t*-test relates the value of a *β* regression coefficient to its estimated standard error. From this, a probability value *p* is calculated for each *β* regression coefficient. It gives the significance of the observed effect by the probability that an estimated *β* value is decided to be non-zero, whereas the true value is zero. Lower *p*-values correspond to a lower probability of erroneous assignment of an effect, and thereby a high statistical significance of the observed effect. A *p*-value < 0.05 corresponding to a confidence of 95% is typically used as indicator of statistical significance. The *p*-values *p* = 0.000 (equivalent to *p* < 0.0005) obtained for most of the hypotheses of our OCM example corresponds to a confidence exceeding 99.95% that two compared catalyst groups are indeed distinctly different in their C_2_ yield. Supplementary Note 8 provides details on the employed regression analysis and significance testing. Supplementary Note 9 illustrates the results of a typical regression analysis exemplarily for the tested OCM hypothesis 1 with respect to group assignment, pressure ratio, temperature, *β* values, *p*-values, and property distributions.

### Estimation strategy

The estimation strategy evaluates four different property–performance hypotheses labeled (1, 2, 3, 4) that divide the initial OCM data successively into smaller subgroups (1a, 1b,…). All individual hypotheses are then assembled into one overall property–performance model (Figs. 3 and 4). The best-performing property group is identified and compared to each other subgroup in terms of *Y*_C2_.

### Robustness

The robustness tests for the final model varied systematically the threshold values of important descriptor values (4× wt% of support oxide (Supplementary Note 14a–d), 3× Tammann factors (Supplementary Note 14e–g), 4× offsets for carbonate stability (Supplementary Note 14h–k)). Moreover, the regression analysis was performed also without reaction-condition compensation (Supplementary Note 15), and with a frequency-weighted multiple regression that accounts for the different frequency of elemental catalyst composition reported in the database (Supplementary Note 16). Moreover, the regression was performed with ln(*Y*_C2_) as independent variable instead of *Y*_C2_, (Supplementary Note 17) and using a so-called robust regression approach that reduces the influence of outliers in the data (Supplementary Note 18).

### Result presentation

The regression results are presented in terms of the number of catalysts assigned to each group (*N*), the effective (corrected) difference in C_2_ yield (regression coefficient *β*_group_, in mol% C_2_) between two compared groups along with the *p*-value corresponding to the regression coefficients (three significant digits). Moreover, the arithmetic mean of uncorrected C_2_ yield (${\bar{{Y}}}$, in mol% C_2_) and corrected (${\bar{{Y}}}$_corr_) are reported along with the corresponding standard deviation. The presented diagrams display for each subgroup the density of yield observations vs. the uncorrected C_2_ yields (see Supplementary Note 12 for details on the calculation of density plots via Epanechnikov kernel density). Supplementary Data 5 (20160713_list_cat-combination_property-groups_.xlsx) provides for the final model a full list of catalyst compositions (i.e., cation combinations) contained in each property group.

### Catalytic testing and experimental validation

Catalytic tests were performed to validate the main findings. Alumina-supported catalysts containing similar loadings of the precursor of either one alkali element (Li, Na, K, Rb, Cs) or one alkaline earth element (Mg, Ca, Sr, Ba) were prepared via impregnation of the corresponding carbonate or acetate onto the same pre-calcined alumina support. The employed γ-Al_2_O_3_ support was supplied by Südchemie/Clariant as pellets (Al_2_O_3_-100), which were ground, sieved to a size fraction of 200–500 µm, and calcined for 12 h at 800 °C in air. The support was then loaded via wet impregnation with aqueous solutions (alkali carbonates, alkaline earth acetates) and dried at 60 and 150 °C under vacuum, i.e., following a procedure adapted from Kusche et al.^69^ Catalyst loadings amounted to 23.1 wt% of the employed salt in anhydrous state (alkali elements: carbonates; alkaline earth elements: acetates).

OCM catalytic tests were performed in a parallel fixed bed reactor featuring 48 quartz tube reactors with 4 mm inner diameter. Each tube carried 50 mg catalyst powder 300 mg of pre-calcined SiC granules placed upstream to ensure efficient feed preheating. Catalytic performance was measured in 25 K intervals (450 °C → 5 K/min ramp → 475 °C,…) up to 850 °C, holding each temperature to analyze product composition via GC^14^. OCM testing was performed with two different feed compositions, i.e., CH_4_/O_2_/N_2_ (26.2:14.8:59.0) and CH_4_/O_2_/N_2_/CO_2_ (26.2:14.8:3.0:56.0), using fresh catalysts for each run and a flow rate of 14.7 Nml/min per reactor channel.

## Supplementary information

Supplementary Information

Description of Additional Supplementary Files

Supplementary Data 1

Supplementary Data 2

Supplementary Data 3

Supplementary Data 4

Supplementary Data 5

## Data Availability

All data employed in this work are available in the Supplementary Files.
