# Supplementary material for: A meta-analysis of catalytic literature data reveals property-performance correlations for the OCM reaction
Source: Nat Commun. 2019 Jan 25;10:441. doi: 10.1038/s41467-019-08325-8 (PMC6347636; doi:10.1038/s41467-019-08325-8)
Supplement: Supplementary file 1 — Supplementary Information [file 41467_2019_8325_MOESM1_ESM.pdf]

A meta-analysis of catalytic literature data reveals property-performance correlations for the OCM reaction

Schmack et al.

Supporting Information

Contents

Supplementary Note 1 OCM data and statistical analysis by Zavyalova et al. 2

Supplementary Note 2 OCM performance prediction and experimental validation based on statistical analysis by Kondratenko et al. 5

Supplementary Note 3 Corrections and changes to the OCM dataset 6

Supplementary Note 4 Corrected dataset employed in our analysis 8

Supplementary Note 5 Element property table 9

Supplementary Note 6 Descriptor rules for elemental properties 10

Supplementary Note 7 Sorting rules for catalyst property groups 12

Supplementary Note 8 Applied regression model and testing for statistical significance 13

Supplementary Note 9 Typical regression parameters and results 15

Supplementary Note 10 Number of observations of cation combinations within property groups 17

Supplementary Note 11a Final model: Matrix of β_{group} regression coefficients and their p-values 18

Supplementary Note 11b Final model: Matrix of β_T regression coefficients and their p-values 20

Supplementary Note 11c Final model: Matrix of $\beta_{CH4/O2}$ regression coefficients and their p-values 21

Supplementary Note 12 Generation of density plots of Y_{C2} via Epanechnikov kernel density function 22

Supplementary Note 13 Hypothesis 4: Frequent cation combinations, yield densities and regression results 23

Supplementary Note 14 Robustness test: Variation of descriptor rule parameters for definition of property groups 24

Supplementary Note 15 Robustness test: Regression without compensation for reaction conditions . 36

Supplementary Note 16 Robustness test: Regression with weighting of similar catalyst compositions37

Supplementary Note 17 Robustness test: Regression towards $ln(Y_{C2})$ as independent variable 38

Supplementary Note 18 Robustness test: Robust regression 39

Supplementary Note 19 Preparation and testing of Al_2O_3 supported catalysts 40

Supplementary Note 20 Experimental C_2 Yield distributions for typical OCM catalysts Li/MgO and Mn-Na-W/SiO_2 41

Supplementary References 42

Supplementary Note 1 OCM data and statistical analysis by Zavyalova et al.

In a landmark publication, Zavyalova et al.[1] collected a large set of historical OCM data from literature published between 1982 and 2009. A total number of 1866 observations from 421 referenced publications (papers, patents, PhD theses) were collected. The dataset was made publically available under http://www.fhi-berlin.mpg.de/acnew/department/pages/ocmdata.html.

In the dataset, the catalyst composition is described in terms of the presence and the molar fractions of elements assigned to the categories cation, anion and support, as well as the presence of "promotors" (without molar fraction). The reaction conditions are provided in terms of the partial pressure of methane and oxygen in the reaction feed, total pressure, temperature and contact time. Catalytic performance is described via conversion of methane and oxygen, C_2 selectivity and C_2 yield. Only catalysts that provided all the listed information were included in the data set. Moreover, only catalysts studied in a fixed bed reactor with continuous methane/oxygen co-feed were considered. Supplementary Figure 1 illustrates the dataset as well as the statistical evaluation by Zavyalova et al.

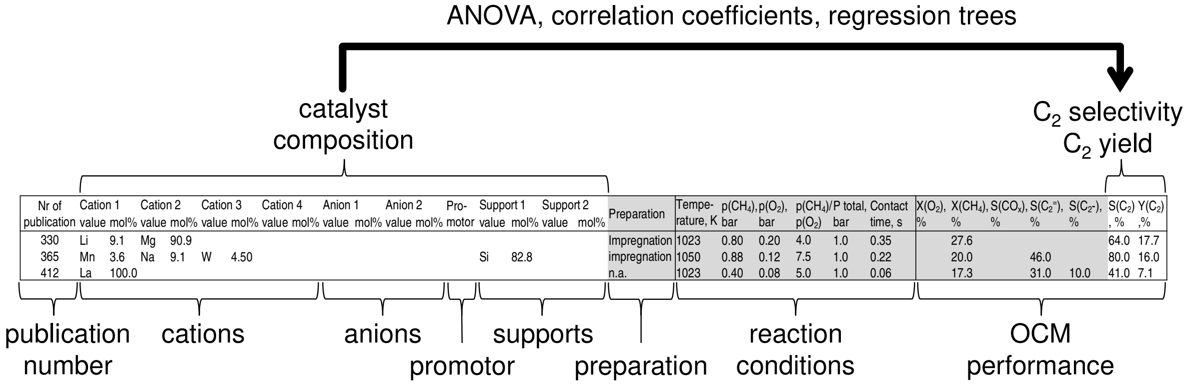

Supplementary Figure 1 Structure of the OCM dataset collected by Zavyalova et al. and implemented data categories.[1] Variables considered in the statistical analysis of Zavyalova et al. are marked by white background color. The arrow illustrates the tested correlations between elemental catalyst composition and indicators of OCM performance.

Zavyalova et al. analysed the dataset by statistical methods in order to identify correlations between elemental catalyst composition and OCM performance. Arithmetic mean values of C_2 selectivity and yield were used to compare the OCM performance of single unsupported oxides that appear at least three times within the dataset. Alkaline earth elements Ca, Sr and Ba as well as lanthanides La, Sm, Nd, Eu, Gd and Yb were found to exhibit relatively high values for C_2 selectivity and yield. However the number of employed observations per element varies, and the variance of performance values for the same element is often times high.

Multiway analysis of variance (ANOVA)[2] was employed to evaluate the effect of the presence or absence of specific elements on catalytic performance. In this case, this method analyses whether a considered element can be left out without significantly changing the variance of the dependent variables C_2 selectivity or yield. To achieve this, a null hypothesis is formulated stating that the variance of the dependent variable does not change if one element is left out. The ANOVA output is a significance level for each element, giving the probability of erroneously rejecting the null hypothesis. This means, a low significance level indicates a strong effect of the considered element on catalytic performance. Zavyalova et al. applied the analysis to either the complete dataset or a subset containing only well-performing catalysts with C_2 yield ≥ 15% and C_2 selectivity ≥ 50%. For example, for alkaline elements Li and Na, as well as for alkaline earth elements Sr and Ba, high significance was found with respect to C_2 selectivity and yield. However, the representative quality of ANOVA results for the group of well-performing is questioned by the authors due to a low number of observations for some of the elements. Furthermore, ANOVA does not indicate whether the effect of an element is positive or negative, which is needed in the context of catalysis.

To establish correlations between the considered variables molar fraction of elements and C_2 selectivity or yield, Zavyalova et al. applied Bravais-Pearson's and Spearman's rank correlation[3]. The Bravais-Pearson correlation evaluates whether a positive or negative linear dependency exists between two variables, and assigns a correlation coefficient. It is defined as the standardized covariance of the two variables and allows values between -1 and 1. The Spearman rank correlation assigns ranks to all appearing values of the two variables. The order of ranks (i.e. lowest to highest appearing value) is then compared for both variables. A correlation coefficient is assigned that evaluates whether a systematic order exists between the ranks of both variables. The coefficient allows values between -1 and 1. In contrast to the Bravais-Pearson coefficient, a linear dependency between both variables is not assumed. Zavyalova et al. show positive correlation coefficients for both C_2 selectivity and yield for alkaline metals (Li, Na, Cs), for alkaline earth metals (Mg, Ca, Sr, Ba), and various other metals (Bi, Mo, Ga, Nd, Re, Yb). However, the authors point out that the obtained correlation coefficient have values below 0.2, indicating that the covariance is small with respect to the variances of the variables. This indicates weak correlations. Additionally, correlation coefficients are generally not suitable for inferring causality[4].

Zavyalova et al. studied the effect of presence or absence of specific elements also using regression trees. In this method, an algorithm was used to split the data subset of well-performing catalysts into two groups maximizing the homogeneity of C_2 selectivity or yield within each of the two groups. To achieve this, the variance of the performance variable within each group is minimized. The criterion to split the dataset was the presence of absence of a considered element. The obtained groups were again subdivided using the same algorithm, until one of the formed groups would contain less than 5% of the observations. However, the regression trees are unstable, as their stepwise development depends strongly on the first split. Starting a regression tree with a different split as starting point would potentially also change the relevant elements for further splitting in the next steps[5] and their effect on the performance variables. In the regression trees, the situation occurs that the same element has opposite effects on performance depending on preceding splits, which indicates the instability of the obtained statements. Furthermore, the difference in homogeneity of the performance variable between two groups formed when splitting the dataset is not documented. Therefore, it cannot be evaluated whether the selected element for splitting is significantly more relevant than any other element. Also, only the mean value of the performance variable is shown for each subgroup, with no information on variance within the subgroup.

As a result of the applied statistical analyses, Zavyalova et al. identified a group of 18 "key elements": Sr, Ba, Mg, Ca; La, Nd, Sm; Ga, Bi, Mo, W, Mn, Re; Li, Na, Cs; F, Cl. For binary combinations of these elements, ANOVA was applied to find those combinations contributing significantly to C_2 selectivity or yield. The extent of their contribution was evaluated by comparing mean values of C_2 selectivity or yield in presence or absence of the respective element combinations, within the group of well performing catalysts. A list of element combinations is given that contribute positively towards C_2 selectivity or yield. Additionally, Zavyalova et al. performed a regression tree analysis using molar fractions of the "key elements" as criteria for group splitting. However the differences in mean C_2 selectivity or yield for the subgroups formed in this regression tree analysis is rather small, and again no information on the statistical significance of the differentiation is given. Some "key elements" were selected multiple times with different molar fractions used as split points. However, no consistent trend between molar fraction and subgroup performance is obtained.

Throughout the statistical analysis performed by Zavyalova et al., the reaction conditions applied to obtain catalytic data are disregarded. This is a severe simplification, as reaction conditions (temperature, methane / oxygen ratio) have a strong influence on OCM performance.
Furthermore, the assignment of elements as either support or active components is inconsistent. For example, in the dataset elements such as Mg, Ca, La appear as both cation components and catalyst support. This is because no objective definition for support components was applied, rather the subjective assignment of the various authors referenced in the dataset was adopted.

The results of the statistical evaluation by Zavyalova et al. are correlations between elemental catalyst composition and OCM performance. From this, a group of “key elements” that positively influence the catalyst performance is derived. However, no direct correlations between catalyst properties and performance are obtained. The catalyst properties discussed for high-performance OCM catalysts are not based on the results of the statistical analysis. It is merely pointed out that some of the “key elements” exhibit strong basicity as a property. One of the main conclusions resulting from the statistical evaluation by Zavyalova et al. is that in order to obtain advanced knowledge, it is required to consider catalyst properties rather than only elemental compositions.

Supplementary Note 2 OCM performance prediction and experimental validation based on statistical analysis by Kondratenko et al.

A work by Kondratenko et al.[6] attempted to validate the identified correlations between elemental catalyst composition and OCM performance reported by Zavyalova et al. and to evaluate the potential of applying statistical models based on the OCM dataset to predict OCM performance of multicomponent materials. To achieve this, the authors compared predicted C_2 yields derived from neural networks with experimentally measured C_2 yields.

The employed clustered radial basis function networks were trained by minimizing various error models. The data used for training and prediction was a subset of the OCM dataset that consisted of catalysts containing only components from a pool of nine elements (Li, Na, Cs, Mg, Sr, Ba, La, Mn, W). The independent variables were the molar fractions of the elements and the dependent variable was the C_2 yield. Reaction conditions (temperature, methane / oxygen ratio) were not regarded in the model. To predict C_2 yields, weighted averages calculated by neural networks distinguished in model details were employed. The C_2 yields of catalysts with 42 different ternary element combinations were predicted. For each ternary combination, the element molar fractions were chosen in a way that the predicted C_2 yield was maximized. For the experimental study, 42 catalysts with different ternary element combinations were synthesized using incipient wetness impregnation. OCM performance of the catalysts were tested in a parallel reactor.

The predicted and experimental C_2 yields show noticeable discrepancies. This is also indicated by a significance test comparing the distribution of predicted and experimental C_2 yields. For some catalyst systems, the null hypothesis of equal yield distribution can be rejected with high significance. For other catalyst systems, the null hypothesis cannot be rejected on a typical significance level of 5%. However, this does not necessarily indicate equal yield distribution of predicted and experimental C_2 yields, as the applied significance test generally can only reject, but not confirm the null hypothesis.
The authors discuss that reasons for the discrepancies can either be inappropriate models or strong deviation in the data used for model training. The authors show by comparing the extent of variation with model error that the employed models are appropriate for most of the catalyst systems. This means the main reason for discrepancy of predicted an experimental C_2 yields are deviations within the dataset. This is plausible, as the performance data in the OCM dataset were obtained with strongly varied reaction conditions. The reaction conditions have a strong influence on the catalyst structure and properties (phases present, catalyst surface, adsorbed gas species, reaction kinetics), as well as on overall catalytic performance. These effects were ignored, as only elemental catalyst composition was used in the modelling.

Supplementary Note 3 Corrections and changes to the OCM dataset

Our analysis of the original OCM dataset of Zavyalova et al. (1866 catalysts) indicated some errors in the data as well as the presence of outliers, i.e. highly untypical experimental conditions. We re-evaluated the corresponding original literature and either corrected the data (147 catalysts) or removed the complete respective catalyst entry from the dataset (64 catalysts) prior to our statistical analysis due to:

- Duplicate entries (i.e. the exact same observation was reported via different publications)
- Missing information (i.e. temperature, CH_4 / O_2 partial pressure)
- Errors in the data (i.e. elemental composition, temperature not consistent with the original report)
- Reactor design or operation not consistent with the stated criteria (i.e. sequential oxygen feed instead of continuous co-feed)
- Extreme and highly unusual values for feed composition (i.e. CH_4 / O_2 ratios > 40, addition of H_2) or contact time (i.e. $\tau < 0.01$ s or $\tau > 200$ s)

The following tables lists all the corrections and changes that we applied to the original OCM dataset. The columns "Publication number" and "ID number" identify the respective catalyst entry in the naming convention of the original dataset. The "publication number" is a number that identifies the respective publication; ID number *n* addresses the *n*th included observation from the respective publication.

Supplementary Table 3a, catalyst entries that were removed from the dataset

Reason for change	Publication number	ID (observation number in publication)	Excluded from dataset	Changed	Detailed information
Duplicated observations	7	12	X		see pub 7 ID 6
	23	5-6	X		see pub 23 ID 1-2
	23	7	X		see pub 24 ID 1
	25	1-9	X		see pub 24 ID 2 - 10
	74	1	X		see pub 52 ID 1
	109	2	X		see pub 109 ID 1
	136	1	X		see pub 90 ID 2
	141	1	X		see pub 71 ID 1
	141	2-3	X		see pub 108 ID 1 - 2
	141	4-12	X		see pub 20 ID 25 - 33
	141	14	X		see pub 140 ID 9
	211	1	X		see pub 210 ID 1
	404	4-6	X		see pub 420 ID 1, 3 - 4
	417	1-3	X		see pub 149 ID 1 - 3
	420	7-8	X		see pub 404 ID 1 - 2
Observation missing in paper	41	2-3	X		no catalysts containing Sr or Ba documented in original paper
Wrong elemental categories	45	4	X		material is BN. no cations, no supports
Feed composition or feeding mode not consistent with required criteria	252	1	X		no oxygen in feed
	255	1-2	X		sequential oxygen feed
	263	1-4	X		no operating temperature documented
	379	1	X		hydrogen in feed
	397	1-15	X		carbon dioxide in feed as oxidizing agent, p_{CH4} / p_{O2} =100

Corrections and changes to the OCM dataset, continued

Supplementary Table 3b, catalyst entries that were corrected in the derived dataset

Reason for change	Publication number	ID (observation number in publication)	Excluded from dataset	Changed	Detailed information
**Error in elemental composition**	7	22		X	Ag instead of Ar, typo
	29	1		X	mol% cation1 to 20% instead of 50%, mol% cation2 to 80% instead of 50%
	52	1		X	mol% cation1 to 4.5%, mol% cation2 to 79.2%, mol% cation3 to 16.3%
**Error in contact time, now recalculated from original data**	29	1		X	τ to 0.04s instead of 0.002s
	30	1		X	τ to 0.012s instead of 0.001s
	222	1-9		X	τ to 0.02s instead of 0.000001s
	223	1-4		X	τ to 0.05s instead of 0.015s
	257	1-3		X	τ to 0.308s instead of 311,785s
	267	1-7		X	τ to 2.7s instead of 9,000s
	408	1-10		X	τ to 0.3s added
**Error in partial pressures**	143	1-3		X	p_{CH4} to 0.8 bar, p_{O2} to 0.2 bar, p_{CH4}/p_{O2} to 4.0
	144	1		X	p_{CH4} to 0.286 bar, p_{O2} to 0.095 bar, p_{CH4}/p_{O2} to 3.0105
	4	7		X	p_{CH4} to 0.65 bar, p_{CH4}/p_{O2} to 8.1 , typo
**Error in temperature**	4	1-47		X	added 273K to the temperature value, temperature was documented in °C
	36	14		X	added 273K to the temperature value, temperature was documented in °C
	105	1-2		X	added 273K to the temperature value, temperature was documented in °C
	181	1		X	changed to 1173K
	220	1-2		X	changed to 1023K
**Error in performance data**	7	1-38		X	S_{C2} were incremental numbers, recalculated $S_{C2}=S_{ethene}+S_{ethane}$ Y_{C2} adjusted accordingly
	7	18		X	Performance data of wrong catalyst, changed according to original publication X_{CH4}=20.6%, S_{ethene}=28.0%, S_{ethane}=35.2%, S_{C2}=63.2%, Y_{C2}=13.2%
**Total**			64	147	

Supplementary Note 4 Corrected dataset employed in our analysis

The dataset resulting from the changes and corrections (see Supplementary Note 3) contains 1802 catalyst entries. Supplementary Figure 4 illustrates the corrected dataset. Variables with white background colour were employed our current statistical evaluation. The complete corrected dataset (Microsoft Excel) is provided in the file in “20160720_corrected-dataset.xls”.

The original work of Zavyalova et al. assigned each element contained in a catalyst to a fixed category of either “Cation”, “Anion”, “Promotor” or “Support” based on the authors intuition and experience. We kept these categories in the corrected dataset structure in order to remain consistent with the original dataset. However, these pre-assigned categories were not used in our statistical evaluation. Instead, respective catalyst functions were assigned via objective and quantitative descriptor and sorting rules (see Figure 1 in the main paper).

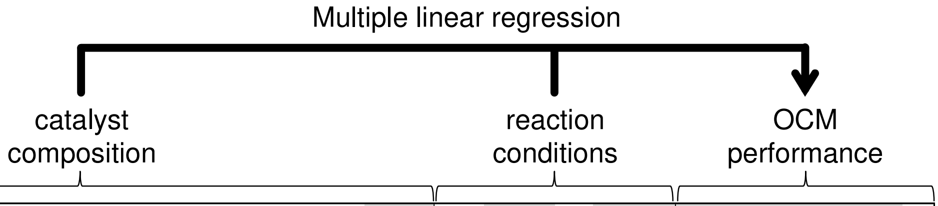

Catalyst Nr	Nr of publication	Cation 1	Cation 1 mol%	Cation 2	Cation 2 mol%	Cation 3	Cation 3 mol%	Cation 4	Cation 4 mol%	Anion 1	Anion 1 mol%	Anion 2	Anion 2 mol%	Promotor	Support 1	Support 1 mol%	Support 2	Support 2 mol%	Preparation	Temperature, K	$p(CH_4)$, bar	$p(O_2)$, bar	$p(CH_4)/p(O_2)$	P total, bar	Contact time, s	$X(O_2)$, %	$X(CH_4)$, %	$S(CO_x)$, %	$S(C_2^=)$, %	$S(C_2-)$, %	$S(C_2)$, %	$Y(C_2)$, %
1353	330	Li	9.1	Mg	90.9														Impregnation	1023	0.80	0.20	4.0	1.0	0.35		27.6				64.0	17.7
1532	365	Mn	3.6	Na	9.1	W	4.5								Si	82.8			impregnation	1050	0.88	0.12	7.5	1.0	0.22		20.0		46.0		80.0	16.0
1806	412	La	100.0																n.a.	1023	0.40	0.08	5.0	1.0	0.06		17.3		31.0	10.0	41.0	7.1

Supplementary Figure 4 Illustration of the structure of the corrected OCM dataset used in this work. Input and target variables employed in statistical evaluation in this work connected by arrows. Variables not employed in statistical evaluation shown in grey.

Supplementary Note 5 Element property table

Basic physical and chemical properties of chemical elements and their compounds were assembled from literature as well as own measurements and compiled in a "property table". Supplementary Figure 5 illustrates the structure and content of the table. The complete table of element properties (Microsoft Excel) is provided as “20160811_element-properties.xls”. The element properties are employed in the descriptor rules to assign descriptors for more complex properties to catalyst compounds.

Element	Atomic number	1st main group	2nd main group	3rd main group	4th main group	5th main group	anion	transi-tion metal	rare earth	Formation of carbonate possible	Oxidation number	Molar mass of element	Molar mass of oxide, per cation	Melting point of chemically most stable oxide /K	Decomposition temperature of carbonate towards oxide /°C
**Li**	3	1	0	0	0	0	0	0	0	yes	1	6.94	14.94	1834	722
**Na**	11	1	0	0	0	0	0	0	0	yes	1	22.98	30.98	1405	910
**Mg**	12	0	1	0	0	0	0	0	0	yes	2	24.31	40.31	3105	469
**Al**	13	0	0	1	0	0	0	0	0	no	3	26.98	50.97	2327	
**Si**	14	0	0	0	1	0	0	0	0	no	4	28.09	60.07	1996	

Supplementary Figure 5 Structure and content of the compiled element property table. The contained element properties can be used via descriptor rules in order to calculate and assign descriptors to a catalyst that reflect its chemical or physical properties based on its elemental composition.

Columns 1 to 10 of the table are self-explaining and reflect the elements name, atomic number and position in the periodic table of elements.

The ability to form a carbonate was assigned based on information available from literature. For example, Si does not form a carbonate under normal conditions[7].

For each cation, the oxidation number and mass per cation is provided for the most thermally stable oxide known for the respective element.

The thermal stability of the most stable oxide with respect to sintering and associated loss of surface area was evaluated via Tammann temperature[8]. The Tamman temperature can be calculated from the oxides melting point (in K) multiplied by a prefeactor. The property table therefore lists the melting temperature of the respective stable oxide and provides the corresponding literature reference. Tamman temperatures were calculated in our analysis via descriptor rules employing different prefactors (see e.g. Supplementary Note 14).

One out of several possible indicators for the basicity of an oxide is the thermal stability of the corresponding carbonate. The thermal stability of the carbonate can be derived from its decomposition temperature, which can be measured experimentally via the mass loss in a thermogravimetric analysis. We measured the respective temperature at which the carbonates of alkali elements (Li, Na, K, Rb, Cs) decompose into the corresponding oxide via TGA (10 K/min, Ar flow). Where possible, the intersection of tangent lines through the inflection points of the mass loss curve was used to derive the decomposition temperature. In the case of multiple decomposition steps (i.e. lanthanide carbonates), the last decomposition step was evaluated. All other carbonate decompositions temperatures were taken from literature as indicated for each element directly inside the element-property table.

Supplementary Note 6 Descriptor rules for elemental properties

The descriptor rules are employed to assign more complex physicochemical properties to possible catalyst compounds based on catalyst composition and element properties. This results in a table with property descriptors for each catalyst compound. Supplementary Figure 6 illustrates for a small subsection the structure of the descriptor table. The complete descriptor table (Microsoft Excel) is provided in “20160729b_corrected_preprocessed_dataset_descriptors.xlsx”.

num	publication	cation_support_combis	cation1_is_support	cation2_is_support	cation3_is_support	cation4_is_support	support1_is_support	support2_is_support	cation1_is_carbonate	cation2_is_carbonate	cation3_is_carbonate	cation4_is_carbonate	support1_is_carbonate	support2_is_carbonate	cation1_is_stabcarb	cation2_is_stabcarb	cation3_is_stabcarb	cation4_is_stabcarb	support1_is_stabcarb	support2_is_stabcarb
1353	330	Li_Mg	0	1	0	0	0	0	1	1	0	0	0	0	1	0	0	0	0	0
1532	365	Mn_Na_Si_W	0	0	0	0	1	0	1	1	0	0	0	0	0	1	0	0	0	0
1806	412	La	1	0	0	0	0	0	1	0	0	0	0	0	1	0	0	0	0	0

Supplementary Figure 6 Illustration of the structure of the descriptor table computed via descriptor rules. The shown columns indicate for each catalyst (columns 1…3) contained in the dataset and for eacht contained element e.g. if a present cation could be considered to act as a support ("is_support"), is able to form a carbonate ("is_carbonate") and if the carbonate is thermally stable at the respective studied OCM temperature ("is_stabcarb").

The ability of a catalyst component to form a carbonate aims to obtain a measure of its basicity. This descriptor rule is fulfilled when a component can form a carbonate, i.e. a carbonate has been reported in literature (see Supplementary Note 5, element property table).

The role of a catalyst support is to provide sufficient surface area for interaction between the catalyst and the gas phase. To reflect this functionality two criteria were defined that a component that could be considered as a support had to fulfil: 1) sufficiently weight fraction in the catalyst and 2) sufficient thermal stability under reaction conditions.

For criterion 1), the support component must be present in sufficiently high quantity within the catalyst composition in order to provide enough material to disperse the other catalyst components. The weight fraction of an oxide component proved to be a suitable representation of the material amount. Equation (1) defines the weight fraction criterion for a component i to fulfil the support function.

$$w_{oxide,i} \geq 50\% \qquad (1)$$

Setting the required weight fraction to 50% in a typical analysis ensured that the support function could be assigned to only one component of the catalyst. However, the robustness tests reported in section Supplementary Note 14 demonstrate also the results for a variation of this parameter.

For criterion 2), a support should be thermally stable to keep its surface area under reaction conditions. The Tammann temperature[8] describes the onset of sintering and loss of surface area. The Tammann temperature is often calculated via multiplication of a compounds melting point with a prefactor. However, there is no general agreement of the exact general value of this Tamman factor. typical values provided in literature range from 0.37[9-11] to 0.75[12-13]. We used a value of 0.60 as criterion for all oxides in the final model, which is close to the value for typical support oxides such as Al_2O_3 and SiO_2[14]. Moreover, the Tammann factor was also subject to a parameter variation (see robustness tests in Supplementary Note 14 for details).

The respective descriptor rule compared the Tamman temperature assigned to the oxide of a catalyst component to the temperature of the corresponding OCM measurement as reported in the dataset. A component was therefore considered to provide a thermally stable support if the respective OCM temperature was lower than a threshold value derived from the oxides melting point. However, the same component may not fulfil the stability criterion if a catalyst was measured at higher OCM temperatures. Formula (2) defines the thermal stability criterion for a component i to fulfil the support function.

$$T_{melt,oxide} \times 0.60 > T_{measurement,i} \qquad (2)$$

A catalyst component was credited with a possible support function if at least one component of the catalyst fulfilled both the weight fraction criterion (1) and the thermal stability criterion (2).

The ability of a catalyst component to form a thermodynamically stable carbonate also at the typically rather high temperatures of the OCM reaction is one possible indicator for strong basicity of a catalyst component. Moreover, if a carbonate is potentially present under OCM reaction conditions, it has the possibility to directly contribute to the overall reactivity of the catalyst.

To estimate whether a carbonate can be stable under the respective OCM conditions, the decomposition temperature of the carbonates that can be formed from the elements contained in a catalyst were individually compared to the respective temperature of OCM measurement for a given catalyst. That means that the same carbonate may be considered thermodynamically stable or not, depending on the temperature employed for the reported OCM experiment.

However, the OCM reaction can form a significant amount of CO_2 as a reaction byproduct. The CO_2 enters a chemical equilibrium between oxide and carbonate, thus stabilizing the carbonate against decomposition. Moreover, the carbonates decomposition temperature in TGA analysis describes only the onset of mass loss, and complete decomposition may only be obtained at higher temperatures. To account for these effects, an offset was introduced when comparing carbonate decomposition and measurement temperature, as shown in formula (3). The value was set to 100 K for the final model. However, other values were tested systematically (see robustness tests in Supplementary Note 14).

$$T_{OCM\ measurement,i} < T_{carbonate\ decomposition} + 100 \qquad (3)$$

If the component of a catalyst with the highest carbonate decomposition temperature fulfils the criterion given by formula (3), that component is assigned the property of the thermodynamically stable carbonate.

Supplementary Note 7 Sorting rules for catalyst property groups

Sorting rules were applied in order to assign catalysts of similar properties into so-called property groups based on the specific formulation of a chemical hypothesis. The sorting results in a table with assigned property groups for each catalyst. Supplementary Figure 7 illustrates the resulting table. The complete table of property groups (Microsoft Excel) is provided for the final model in "20160729b_corrected_preprocessed_dataset_property_groups.xlsx".

Hypothesis			(1)	(2)				(3)		(4)							
num	publication	cation_support_combis	carbonate_formation	un-supported	supported	self_supported	not_assigned	carbonate_supported	inert_supported	unsup-ported_stable	unsup-ported_unstable	carbonate_supported_stable	carbonate_supported_unstable	inert_supported_stable	inert_supported_unstable	self_supported_stable	self_supported_unstable
1353	330	Li_Mg	1	0	1	0	0	1	0	0	0	1	0	0	0	0	0
1532	365	Mn_Na_Si_W	1	0	1	0	0	0	1	0	0	0	0	1	0	0	0
1806	412	La	1	0	0	1	0	0	0	0	0	0	0	0	0	1	0

Supplementary Figure 7 Illustration of the table of property groups. Group assignments are indicated by a value "1" in the corresponding column.

For hypothesis 1, if a catalyst contains at least one element that can form a carbonate, it is located in the group "Can form a carbonate". Otherwise it is assigned to the group "Cannot form a carbonate".

In hypothesis 2, only catalysts from the "Can form carbonate" group from the first hypothesis were further evaluated. If assigned to the "Unsupported" group, none of the catalyst components fulfilled the support function criteria. In the "Supported" group, one component fulfilled the support function and additionally, another component besides the support was able to form a carbonate. The "Self-Supported" group was defined as catalysts that contain only one component, with this component fulfilling also the support function criteria. Catalysts in the "Not assigned" group did not fit in any of the three previously described groups. This is e.g. the case whenever there is one support component, but none of the other components has the ability to form a carbonate.

In hypothesis 3, catalysts from the "Supported" group were further analyzed. If the support component had the ability to form a carbonate, the catalyst was placed in the group "Carbonate supported". Otherwise the support component was assumed not to be able to form a carbonate, and the catalyst was assigned to the group "Oxide supported".

Hypothesis 4 was applied to all catalysts contained in the property groups deduced by hypothesis 2 and 3. In hypothesis 4, catalysts were further assigned to subgroups based on the stability of the carbonate with the highest decomposition temperature among all components of the catalyst. If the formation of a carbonate is thermodynamically possible for this component, the catalyst is located in the respective subgroup "At least one thermodynamically stable carbonate". Otherwise, the catalyst was assigned to the subgroup "No thermodynamically stable carbonate".

Supplementary Note 8 Applied regression model and testing for statistical significance

The presented approach divides the catalysts contained in the dataset into groups based on sorting rules that use catalyst properties a criterion for the group assignment. Then, two resulting groups are selected and compared to each other with respect to differences in OCM performance between the two groups. The comparison employs a multiple regression analysis that can account also to some extent for the scatter in individual experimental conditions (temperature, p_{CH4}/p_{O2}) reported for each catalyst.

The choice of a target variable that adequately represents "OCM performance" for a given catalyst is not trivial. The final model therefore employs the C_2 yield as performance indicator. The dataset provided for each catalyst the conversion of methane and oxygen, selectivity to CO_X, ethane, ethylene, and the sum of ethane and ethylene as well as the yield of C_2 products. The available parameters do not allow the calculation of a (kinetic) rate of C_2 formation for several reasons (data not measured under isothermal conditions / kinetic regime / differential reactor, etc …). Using methane conversion would not allow to distinguish between the desired formation of C_2 products and undesired methane combustion. Selectivity is also a poor measure due to the fact that methane conversion and the ratio of CH_4 / O_2 partial pressure in the feed vary widely between different catalysts. Using Y_{C2} as performance indicator represent a necessary compromise in light of the availability of data. However, also literature typically employs Y_{C2} for a comparison, e.g. as criterion for the economic viability of the OCM process[15,16].

The analysis was always performed by comparing two catalyst groups to each other. The comparison employs a simple equation (eq. 1) that describes Y_{C2} as a function of the group assignment, reaction temperature and the feed pressure ratio p_{CH4}/p_{O2}. A dummy variable $D_{group,i}$ reflects if a catalyst belongs to one ("0") or the other group ("1"). The comparison is complicated by the fact that the catalytic tests reported in the dataset were not conducted under the same reaction conditions. However, the employed reaction conditions influence the obtained C_2 yield. To compensate for this effect, the individual reaction conditions temperature and CH_4 / O_2 partial pressure ratio also enter the applied regression as variables. Due to a lack of better correlations that describe the complex reaction kinetics and supported by the distribution of experimental results (Figure 2) both effects are approximated by linear terms.

$$Y_i = \beta_0 + \beta_{group} \times D_{group,i} + \beta_T \times \Delta T_i + \beta_{CH4/O2} \times \Delta p_{CH4/O2,i} + U_i \qquad (1)$$

In equation (1), each catalyst i in the dataset has the following variables:
- Y_i is the C_2 yield of the catalyst as reported in the dataset and described by three independent variables $D_{group,i}$, ΔT_i and $\Delta p_{CH4/O2,i}$.
- $D_{group,i}$ is a dummy variable with value either 0 or 1 describing the position of catalyst i in one of the two catalyst groups that are compared.
- ΔT_i is the difference between the measurement temperature applied to the catalyst i and the mean measurement temperature of all catalysts in the dataset.
- $p_{CH4/O2}$ is the CH_4 / O_2 partial pressure ratio. Accordingly, $\Delta p_{CH4/O2,i}$ is the difference of measurement ratio applied to catalyst i and the mean measurement ratio of all catalysts in the dataset.

In equation (1), each independent variable ($D_{group,i}$, ΔT_i and $\Delta p_{CH4/O2,i}$) is multiplied with a corresponding regression coefficient β_{group}, β_T and $\beta_{CH4/O2}$. An additional regression coefficient β_0 represents the default case of a catalyst in the first property group ($D_{group} = 0$) at mean temperature and CH_4 / O_2 partial pressure ratio. U_i represent a residual difference between the C_2 yield for catalyst i calculated via regression vs. the experimental value reported in the dataset. The applied multiple linear regression adjusts for each group comparison the regression coefficients β_0, β_{group}, β_T and $\beta_{CH4/O2}$ in a way that minimizes the sum of the ordinary least squares (OLS)[17] of the residuals U_i.

The obtained β regression coefficient provide valuable quantitative information on the effect of each independent variable on the C_2 yield. β_{group} quantifies the effect of the tested catalyst properties (i.e. group assignment) on OCM performance in terms of the difference in C_2 yield between the two groups. However, it should be kept in mind that β_{group} is not strictly identical to the difference of reported mean C_2 yield for the two groups due to the fact that the regression equation includes a compensation for different reaction parameters for each catalyst (see e.g. Supplementary Note 14 for a robustness test that does compares the regression results with and without correction for differences in T and p_{CH4}/p_{O2}).

Moreover, the regression coefficients β_T and $\beta_{CH4/O2}$ provide a rough estimate to which extent the reaction parameters temperature and CH_4 / O_2 ratio contribute to the observed differences in C_2 yield. The typically obtained positive values in β_T and negative values in $\beta_{CH4/O2}$ reflect the general observation that high temperatures and high oxygen partial pressures often lead to increased C_2 yields (see e.g. Supplementary Note 9).

The statistical significance of information derived from each β regression coefficient was evaluated by a T-test. The T-test relates the derived value of the respective β regression coefficient to its standard error estimated from the observations contained in the evaluated subsection of the dataset (i.e. the compared groups). From this, the p-value for each β regression coefficient is calculated. The p value describes the significance of the observed effect (e.g. a difference in Y_{C2} between the two compared groups) by the probability that an estimated β value is concluded to be different from zero, whereas the true value is zero. A low p-value indicates a low probability of erroneous assignment of an effect, and therefore corresponds to a high statistical significance of the observed effect. Usually a confidence of at least 95% ($p \leq 0.050$) is chosen as requirement for sufficient statistical significance.

Supplementary Note 9 Typical regression parameters and results

Equation (1) describes the regression typically used for the comparison of the OCM performance of two property groups with implemented compensation for reaction conditions.

$$Y_i = \beta_0 + \beta_{group} \times D_{group,i} + \beta_T \times \Delta T_i + \beta_{CH4/O2} \times \Delta p_{CH4/O2,i} + U_i \qquad (1)$$

Each regression coefficient ($\beta_{group,}$, β_T, $\beta_{CH4/O2}$) represents the effect of the corresponding independent variable on the dependent variable (C_2 yield), while taking all other independent variables into account. These effects can be visualised via the respective component plus residual ("CPR") plot. The CPR values represent the remaining variance of the dependent variable (C_2 yield) to be explained by the considered independent variable. The component plus residual values for the independent variables are calculated according to equation (2a-c).

$$cpr_{group,i} = Y_i - \beta_0 - \beta_T \times \Delta T_i - \beta_{CH4/O2} \times \Delta p_{CH4/O2,i} = \beta_{group} \times D_{group,i} + U_i \qquad (2a)$$
$$cpr_{T,i} = Y_i - \beta_0 - \beta_{group} \times D_{group,i} - \beta_{CH4/O2} \times \Delta p_{CH4/O2,i} = \beta_T \times \Delta T_i + U_i \qquad (2b)$$
$$cpr_{CH4/O2,i} = Y_i - \beta_0 - \beta_{group} \times D_{group,i} - \beta_T \times \Delta T_i = \beta_{CH4/O2} \times \Delta p_{CH4/O2,i} + U_i \qquad (2c)$$

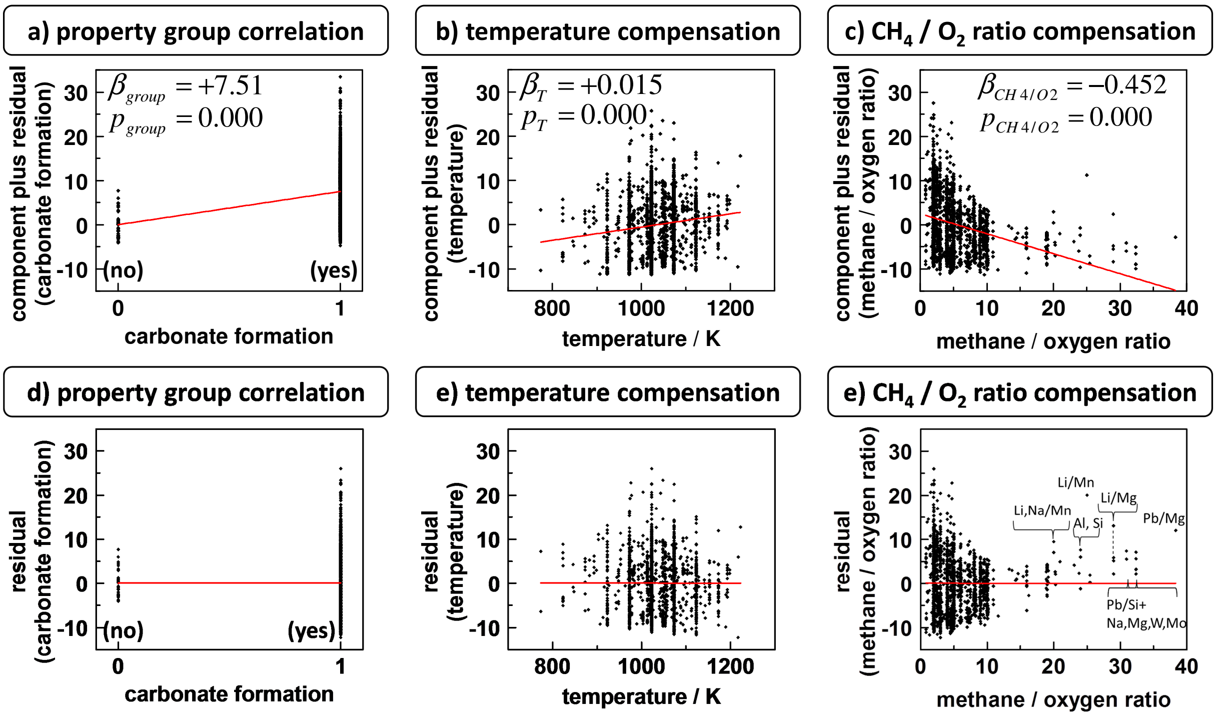

Supplementary Figure 9 Residual plots. a)-c) Component plus residual "CPR" plots for the independent variables property group (group 1a vs 1b), temperature, and CH_4 / O_2 ratio depicted exemplarily for hypothesis 1. Black dots correspond to the experimental values reported in the dataset, i.e. the variance in C_2 yield to be explained by the considered independent variable after accounting for the contributions of the other independent variables. Red lines indicate a linear regression fit to the data. The slopes of the red lines equals the corresponding β regression coefficients. Hence, red lines represent the "effect" on Y_{C2} identified via regression analysis from the experimental data (black symbols) in the (corrected) OCM dataset. d)-f) Residual plots for the independent variables property group (group 1a vs 1b), temperature, and CH_4 / O_2 ratio depicted exemplarily for hypothesis 1. Black dots correspond to the experimental values reported in the dataset, i.e. the variance in C_2 yield to be explained by the considered independent variable after accounting for the contributions of the other independent variables.

The CPR plots are discussed here exemplarily for hypothesis 1 in order to illustrate the general regression procedure and outcome. In hypothesis 1 all catalysts were divided into two property groups, depending on whether they contain at least one element that can form a carbonate (group 1b) or not

(group 1a). Supplementary Figure 9a/b/c shows CPR plots for all three independent variables, i.e. property group, temperature and CH_4 / O_2 ratio corresponding to hypothesis 1.

The value of the regression coefficient $\beta_{group} = +7.62$ indicates the catalysts in the dataset that are able to form a carbonate show on average a C_2 yield that is 7.62% higher than for catalysts that do not contain an element that can form a carbonate. The value $p = 0.000$ indicates a high significance of the correlation. An interpretation of this value in context of OCM is given in the discussion of the first hypothesis.

The obtained regression coefficient for the measurement temperature amounts to $\beta_T = +0.012$. The respective p value amounts to p=0.000, which indicated a high statistical significance. Hence, C_2 yields increase on average by 0.012% per Kelvin, or 1.2% when the measurement temperature is increased by 100 K. This positive correlation relates to the fact that methane conversion typically increases with temperature in OCM and that a high temperature and complete methane conversion also often contribute to a higher C_2 selectivity [18-22]. However, the positive trend indicates by no means that an increased temperature induces for each individual catalyst an increased C_2 yield: It represents only a generally trend observed over many different catalysts contained in the studied dataset.

The regression coefficient obtained for the influence of the pressure ratio CH_4 / O_2 in the feed amounts to $\beta_{CH4/O2} = -0.431$. The respective p value amounts to p=0.000, which indicated a high statistical significance. Hence, a ratio that is increased by 1 via e.g. changing p_{CH4}:p_{O2} from 2:2 to 2:1 results in a C_2 yield that is on average 0.431 percentage points lower when averaged over the studied dataset. This negative correlation can be explained by a low oxygen concentration limiting the conversion of methane. While such a change can increase the C_2 selectivity, the lower methane conversion results in low overall the C_2 yield. Studies that varied the CH_4 / O_2 ratio for various catalysts report consistent observations for individual catalysts[20-22].

The influence of the reaction conditions (expressed as β_T and $\beta_{CH4/O2}$) on C_2 yield shown in Supplementary Figure 9b/c were observed to be qualitatively similar for all other hypotheses tested in this work. The regression coefficients β_T and $\beta_{CH4/O2}$ and respective p values that were obtained for all hypotheses tested with the final model are provided in the Supplementary Notes 11b (β_T) and Supplementary Notes 11c ($\beta_{CH4/O2}$) along with the corresponding group effect (Supplementary Notes 11a, $\beta_{group,}$).

The same data as in Supplementary Figure 9 a/b/c can also be presented as a residual analysis without the components, i.e. values of U_i plotted against the independent variables as shown in Supplementary Figure 9 d/e/f. The slopes of the linear fits become zero and the residuals follow a reasonably random distribution. For catalysts exhibiting noticeable high positive residuals, the compositions are shown. Judging by the compositions, these appear as typical OCM catalysts. Therefore, it can be assumed that the deviation is largely caused by variance in performance results and is not caused by special catalyst properties.

Supplementary Note 10 Number of observations of cation combinations within property groups

The number of literature reports and therefore also the number of corresponding dataset entries varies significantly between very popular catalyst systems (e.g. Li/MgO, 72 observations) and less frequently studied systems (Li/CaO, 27 observations). The number of observations can be deduced by counting the unique cation combinations contained in the dataset. The applied procedure produces cation combinations labelled by e.g. "Li_Mg" for catalyst containing Li and Mg, or "Ca_Pb" for a catalyst containing Ca and Pb, irrespective of the chemical state, phase composition, element concentration etc of the catalyst. Supplementary Figure 10 illustrates the most frequent cation combinations in the different groups produced by the final model. Note that the cation combinations are unique and do not overlap with each other, i.e. all catalyst containing only Na and Ca in combination are assigned to “Ca_Na”, but are not counted for e.g. catalysts that containing only Ca (“Ca”). The complete table of cation combination within all property groups (Microsoft Excel) is provided in the supplemented file “20160713_list_cat-combination_property-groups.xlsx”.

group 0: All catalysts		group 1a: Cannot form a carbonate		group 1b: Can form a carbonate		group 2a: Unsupported		group 2b: Supported		group 2c: Self-supported		group 2d: Not assigned		group 3a: Carbonate supported		group 3b: Oxide supported	
cation combination	number of obs.	cation combination	number of obs.	cation combination	number of obs.	cation combination	number of obs.	cation combination	number of obs.	cation combination	number of obs.	cation combination	number of obs.	cation combination	number of obs.	cation combination	number of obs.
**Total**	**1802**	**Total**	**38**	**Total**	**1764**	**Total**	**197**	**Total**	**1257**	**Total**	**263**	**Total**	**47**	**Total**	**935**	**Total**	**322**
Li_Mg	72	Si	12	Li_Mg	72	Al_Pb	8	Li_Mg	72	La	36	Al_La	4	Li_Mg	72	Mn_Na_Si_W	13
Ca_Na	39	Al	6	Ca_Na	39	Mo_Na	5	Ca_Na	39	Ca	35	Mg_Sn	4	Ca_Na	39	Mn_Na_Si	12
La	36	Si_V	3	La	36	Pb	5	La_Sr	30	Mg	27	Ba_Ti	3	La_Sr	30	Li_Ti	12
Ca	35	Si_W	3	Ca	35	Bi	5	Ca_Li	27	Ba	23	Ca_Si	2	Ca_Li	27	Na_Si_W	10
La_Sr	30	Nb	2	La_Sr	30	Na	5	Ba_La	22	Sr	18	Al_Ba	2	Ba_La	22	Al_Pb	9
Ca_Li	27	Sn	2	Ca_Li	27	K_Sb	5	Ca_Pb	20	Sm	18	Al_Zn	2	Ca_Pb	20	Al_Ba	9

Supplementary Figure 10 Illustration of the structure and content of the table 20160713_list_cat-combination_property-groups.xlsx that lists the number of observations of all cation combinations in the dataset and derived groups.

The number of observations of each unique cation combination can be used to implement weight factors that correct for the differences in observation frequencies. However, a corresponding robustness test as reported in Supplementary Note 16 did not alter the final model in a significant way.

Supplementary Note 11a Final model: Matrix of β_{group} regression coefficients and their p-values

In order to test the proposed models the regression analysis was performed for each possible binary combination of the deduced catalyst property groups. The tree representation in Figure 4 of the main manuscript displays only a fraction of these test results i.e. the 17 most relevant results out of 136 possible regression tests between two subgroups. Supplementary Table 11a presents a comprehensive overview over β_{group} and p-values for all 136 possible binary comparisons. The data is complemented by matrixes Supplementary Table 11b and Supplementary Table 11c which display the corresponding β_T and $\beta_{CH4/O2}$.

To read such a matrix, the two property groups of interest have to be selected by line and column. The thus addressed cell of the table contains the corresponding β_{group} and p-value of the regression. In analogy to the arrows in Fig. 4 in the main article, each regression has a direction. Comparing e.g. group 1b (row) with group 1a (column) indicates a β_{group} of +7.51 and p = 0.000. Hence catalysts in group 1b provide on average a Y_{C2} that is by 7.51 percentage points higher than for group 1a, with a high statistical significance of the deduced effect. Switching rows and columns in this comparison results in the same effect value, just with the opposite sign (-7.51). Redundant entries are provided for clarity in the upper right part of the matrix, marked with grey background. Moreover, results obtained with higher statistical significance ($p < 0.05$) are marked in bold fonts for better readability. The number of catalyst entries is provided for each property group as bold figure located in the first column.

	**0** All catalysts																
		**1a** No carb	**1b** Can form a carbonate														
				**2a** Unsupp.			**2b** Supported							**2c** Self-supp.			**2d** n.a.
								**3a** Carb supp.			**3b** Oxide supp.						
					4a	4b			4c	4d		4e	4f		4g	4h	
**0** All catalysts 1802		+7.32<br>0.000	-0.16<br>0.456	+1.43<br>0.003	+2.46<br>0.004	+0.99<br>0.074	-1.11<br>0.000	-1.94<br>0.000	-0.16<br>0.775	-2.25<br>0.000	+1.33<br>0.001	+5.00<br>0.000	+0.00<br>0.997	+2.64<br>0.000	+2.59<br>0.000	+2.70<br>0.000	+3.22<br>0.001
**1a** Cannot a form carbonate 38	-7.32<br>0.000		-7.51<br>0.000	-6.00<br>0.000	-4.45<br>0.000	-6.46<br>0.000	-8.43<br>0.000	-9.31<br>0.000	-7.59<br>0.000	-9.68<br>0.000	-5.94<br>0.000	-2.59<br>0.000	-7.08<br>0.000	-4.68<br>0.000	-4.69<br>0.000	-4.63<br>0.000	-4.01<br>0.000
**1b** Can form a carbonate 1764	+0.16<br>0.456	+7.51<br>0.000		+1.59<br>0.001	+2.64<br>0.002	+1.14<br>0.038	-0.95<br>0.000	-1.79<br>0.000	+0.05<br>0.925	-2.10<br>0.000	+1.50<br>0.000	+5.19<br>0.000	+0.16<br>0.713	+2.80<br>0.000	+2.77<br>0.000	+2.83<br>0.000	+3.37<br>0.000
**2a** Unsupported 197	-1.43<br>0.003	+6.00<br>0.000	-1.59<br>0.001		+1.47<br>0.081	-0.55<br>0.393	-2.54<br>0.000	-3.42<br>0.000	-1.25<br>0.048	-3.77<br>0.000	-0.13<br>0.831	+3.52<br>0.000	-1.46<br>0.028	+1.19<br>0.012	+1.22<br>0.024	+1.00<br>0.152	+1.51<br>0.110
**4a** No thermodynamically stable carbonate 57	-2.46<br>0.004	+4.45<br>0.000	-2.64<br>0.002	-1.47<br>0.081		-2.04<br>0.026	-3.57<br>0.000	-4.50<br>0.000	-2.84<br>0.000	-4.88<br>0.000	-1.20<br>0.242	+1.87<br>0.017	-2.50<br>0.024	-0.19<br>0.776	-0.15<br>0.832	-0.61<br>0.461	+0.07<br>0.948
**4b** At least one carbonate thermodynamically stable 140	-0.99<br>0.074	+6.46<br>0.000	-1.14<br>0.038	+0.55<br>0.393	+2.04<br>0.026		-2.09<br>0.000	-2.94<br>0.000	-0.65<br>0.353	-3.27<br>0.000	+0.41<br>0.552	+4.04<br>0.000	-0.78<br>0.294	+1.70<br>0.001	+1.69<br>0.004	+1.56<br>0.038	+2.12<br>0.036
**2b** Supported 1257	+1.11<br>0.000	+8.43<br>0.000	+0.95<br>0.000	+2.54<br>0.000	+3.57<br>0.000	+2.09<br>0.000		-0.84<br>0.002	+1.00<br>0.090	-1.15<br>0.000	+2.45<br>0.000	+6.14<br>0.000	+1.14<br>0.016	+3.74<br>0.000	+3.69<br>0.000	+3.76<br>0.000	+4.34<br>0.000
**3a** Carbonate supported 935	+1.94<br>0.000	+9.31<br>0.000	+1.79<br>0.000	+3.42<br>0.000	+4.50<br>0.000	+2.94<br>0.000	+0.84<br>0.002		+1.91<br>0.001	-0.31<br>0.283	+3.30<br>0.000	+7.00<br>0.000	+1.99<br>0.000	+4.60<br>0.000	+4.56<br>0.000	+4.60<br>0.000	+5.16<br>0.000
**4c** No thermodynamically stable carbonate 134	+0.16<br>0.775	+7.59<br>0.000	-0.05<br>0.925	+1.25<br>0.048	+2.84<br>0.000	+0.65<br>0.353	-1.00<br>0.090	-1.91<br>0.001		-2.36<br>0.000	+1.36<br>0.053	+4.98<br>0.000	-0.14<br>0.858	+2.70<br>0.000	+2.73<br>0.000	+2.31<br>0.001	+2.97<br>0.002
**4d** At least one carbonate thermodynamically stable 801	+2.25<br>0.000	+9.68<br>0.000	+2.10<br>0.000	+3.77<br>0.000	+4.88<br>0.000	+3.27<br>0.000	+1.15<br>0.000	+0.31<br>0.283	+2.36<br>0.000		+3.64<br>0.000	+7.37<br>0.000	+2.36<br>0.000	+4.92<br>0.000	+4.90<br>0.000	+4.86<br>0.000	+5.47<br>0.000
**3b** Oxide supported 322	-1.33<br>0.001	+5.94<br>0.000	-1.50<br>0.000	+0.13<br>0.831	+1.20<br>0.242	-0.41<br>0.552	-2.45<br>0.000	-3.30<br>0.000	-1.36<br>0.053	-3.64<br>0.000		+3.73<br>0.000	-1.29<br>0.037	+1.28<br>0.013	+1.17<br>0.055	+1.19<br>0.144	+1.91<br>0.081
**4e** No thermodynamically stable carbonate 86	-5.00<br>0.000	+2.59<br>0.000	-5.19<br>0.000	-3.52<br>0.000	-1.87<br>0.017	-4.04<br>0.000	-6.14<br>0.000	-7.00<br>0.000	-4.98<br>0.000	-7.37<br>0.000	-3.73<br>0.000		-5.11<br>0.000	-2.24<br>0.000	-2.25<br>0.000	-2.25<br>0.001	-1.90<br>0.037
**4f** At least one carbonate thermodynamically stable 236	-0.00<br>0.997	+7.08<br>0.000	-0.16<br>0.713	+1.46<br>0.028	+2.50<br>0.024	+0.78<br>0.294	-1.14<br>0.016	-1.99<br>0.000	+0.14<br>0.858	-2.36<br>0.000	+1.29<br>0.037	+5.11<br>0.000		+2.48<br>0.000	+2.30<br>0.001	+2.02<br>0.020	+3.03<br>0.008
**2c** Self-supported 263	-2.64<br>0.000	+4.68<br>0.000	-2.80<br>0.000	-1.19<br>0.012	+0.19<br>0.776	-1.70<br>0.001	-3.74<br>0.000	-4.60<br>0.000	-2.70<br>0.000	-4.92<br>0.000	-1.28<br>0.013	+2.24<br>0.000	-2.48<br>0.000		-0.03<br>0.945	+0.00<br>0.995	+0.39<br>0.606
**4g** No thermodynamically stable carbonate 173	-2.59<br>0.000	+4.69<br>0.000	-2.77<br>0.000	-1.22<br>0.024	+0.15<br>0.832	-1.69<br>0.004	-3.69<br>0.000	-4.56<br>0.000	-2.73<br>0.000	-4.90<br>0.000	-1.17<br>0.055	+2.25<br>0.000	-2.30<br>0.001	+0.03<br>0.945		+0.05<br>0.933	+0.67<br>0.407
**4h** At least one carbonate thermodynamically stable 90	-2.70<br>0.000	+4.63<br>0.000	-2.83<br>0.000	-1.00<br>0.152	+0.61<br>0.461	-1.56<br>0.038	-3.76<br>0.000	-4.60<br>0.000	-2.31<br>0.001	-4.86<br>0.000	-1.19<br>0.144	+2.25<br>0.001	-2.02<br>0.020	-0.00<br>0.995	-0.05<br>0.933		+0.14<br>0.881
**2d** Not assigned 47	-3.22<br>0.001	+4.01<br>0.000	-3.37<br>0.000	-1.51<br>0.110	-0.07<br>0.948	-2.12<br>0.036	-4.34<br>0.000	-5.16<br>0.000	-2.97<br>0.002	-5.47<br>0.000	-1.91<br>0.081	+1.90<br>0.037	-3.03<br>0.008	-0.39<br>0.606	-0.67<br>0.407	-0.14<br>0.881	

Supplementary Table 11a Matrix of regression results for the final regression model. Each cell provides the β_{group} regression coefficient and p-value corresponding to the comparison between the two selected property groups. Bold entries mark cells with $p < 0.05$. A grey background indicates duplicate entries (opposite sign for the deduced effect). The groups correspond to the final model as presented in Fig. 4 of the main manuscript.

The overwhelming majority of the performed tests proved to be highly significant, with 104 out of 136 binary group comparisons reaching a significance level < 5% ($p < 0.05$) (bold entries in Supplementary Table 11a). Moreover, the test with the highest $\bar{Y}_{C2,\ corr}$ difference can be easily identified via β_{group} values.

The highest significant differences are observed when the group "Cannot form a carbonate" (column 1a) is compared to group 1b ("Can form a carbonate", β_{group} = 7.51), group 2b ("Supported", β_{group} = 8.43), group 3a ("Carbonate supported", β_{group} = 9.31) and group 4d ("At least one carbonate thermodynamically stable, β_{group} = 9.68). It is clear from the results that β_{group} values increase from hypothesis level 1 to 4, i.e with increasing complexity and specificity of the hypothesis expression.

The table can serve as a guideline for experiments that aim to validate or investigate the property-performance relationships indicated by the statistical analysis. One could e.g. select catalysts from two property groups that show a large difference in Y_{C2} and a low p-value, and study these selected catalyst e.g. spectroscopically with respect to the physico-chemical property that distinguishes the two corresponding groups. Such a study (performed preferably in-situ under OCM conditions) could confirm the presence or absence of the respective property.

If, for example, one would chose to study the role of the thermodynamic stability of a carbonate spectroscopically under OCM conditions, selecting oxide supported catalysts from group 4e and 4f (β_{group} = +5.11) would be recommended over carbonate supported (4c/4d, +2.36), unsupported (4a/4b, +2.04) as well as self-supported (4g/4h, -0.05) from a statistical point of view (see β_{group} values in Fig. 4 and Supplementary Table 11a). An experimental evaluation of the influence of the supports ability to form a carbonate could be studied by comparing groups 4c and 4e (+4.89) instead of 3a and 3b (+3.30).

Supplementary Note 11b Final model: Matrix of β_T regression coefficients and their p-values

Each multivariate regression analysis with temperature compensation produces also a matrix of β_T values and the corresponding p values. The matrix resulting for the final model is provided below. The large majority of regression tests indicate a positive and statistically significant influence of temperature on C_2 yield.

Group	N	**0** All catalysts	**1a** No carb	**1b** Can form a carbonate	**2a** Unsupp.	4a	4b	**2b** Supported	**3a** Carb supp.	4c	4d	**3b** Oxide supp.	4e	4f	**2c** Self-supp.	4g	4h	**2d** n.a.
**0** All catalysts	1802		+0.013<br>0.000	+0.015<br>0.000	+0.015<br>0.000	+0.014<br>0.000	+0.014<br>0.000	+0.014<br>0.000	+0.015<br>0.000	+0.015<br>0.000	+0.015<br>0.000	+0.014<br>0.000	+0.014<br>0.000	+0.016<br>0.000	+0.014<br>0.000	+0.014<br>0.000	+0.014<br>0.000	+0.013<br>0.000
**1a** Cannot a form carbonate	38	+0.013<br>0.000		+0.015<br>0.000	+0.019<br>0.003	+0.021<br>0.001	+0.016<br>0.037	+0.015<br>0.000	+0.015<br>0.000	+0.022<br>0.000	+0.018<br>0.000	+0.015<br>0.006	+0.013<br>0.001	+0.023<br>0.001	+0.013<br>0.002	+0.010<br>0.044	+0.012<br>0.018	-0.004<br>0.584
**1b** Can form a carbonate	1764	+0.015<br>0.000	+0.015<br>0.000		+0.016<br>0.000	+0.016<br>0.000	+0.016<br>0.000	+0.015<br>0.000	+0.016<br>0.000	+0.016<br>0.000	+0.017<br>0.000	+0.016<br>0.000	+0.015<br>0.000	+0.017<br>0.000	+0.015<br>0.000	+0.015<br>0.000	+0.016<br>0.000	+0.015<br>0.000
**2a** Unsupported	197	+0.015<br>0.000	+0.019<br>0.003	+0.016<br>0.000		+0.029<br>0.000	+0.027<br>0.000	+0.017<br>0.000	+0.018<br>0.000	+0.028<br>0.000	+0.021<br>0.000	+0.020<br>0.000	+0.020<br>0.000	+0.029<br>0.000	+0.019<br>0.000	+0.019<br>0.000	+0.023<br>0.000	+0.017<br>0.023
**4a** No thermodynamically stable carbonate	57	+0.014<br>0.000	+0.021<br>0.001	+0.016<br>0.000	+0.029<br>0.000		+0.029<br>0.000	+0.016<br>0.000	+0.017<br>0.000	+0.033<br>0.000	+0.021<br>0.000	+0.019<br>0.001	+0.022<br>0.000	+0.030<br>0.000	+0.018<br>0.000	+0.019<br>0.001	+0.025<br>0.000	+0.014<br>0.158
**4b** At least one carbonate thermodynamically stable	140	+0.014<br>0.000	+0.016<br>0.037	+0.016<br>0.000	+0.027<br>0.000	+0.029<br>0.000		+0.016<br>0.000	+0.017<br>0.000	+0.028<br>0.000	+0.021<br>0.000	+0.020<br>0.000	+0.018<br>0.003	+0.031<br>0.000	+0.018<br>0.000	+0.017<br>0.004	+0.023<br>0.002	+0.014<br>0.178
**2b** Supported	1257	+0.014<br>0.000	+0.015<br>0.000	+0.015<br>0.000	+0.017<br>0.000	+0.016<br>0.000	+0.016<br>0.000		+0.016<br>0.000	+0.016<br>0.000	+0.017<br>0.000	+0.016<br>0.000	+0.015<br>0.000	+0.017<br>0.000	+0.015<br>0.000	+0.015<br>0.000	+0.016<br>0.000	+0.014<br>0.000
**3a** Carbonate supported	935	+0.015<br>0.000	+0.015<br>0.000	+0.016<br>0.000	+0.018<br>0.000	+0.017<br>0.000	+0.017<br>0.000	+0.016<br>0.000		+0.018<br>0.000	+0.018<br>0.000	+0.016<br>0.000	+0.016<br>0.000	+0.019<br>0.000	+0.016<br>0.000	+0.016<br>0.000	+0.017<br>0.000	+0.015<br>0.000
**4c** No thermodynamically stable carbonate	134	+0.015<br>0.000	+0.022<br>0.000	+0.016<br>0.000	+0.028<br>0.000	+0.033<br>0.000	+0.028<br>0.000	+0.016<br>0.000	+0.018<br>0.000		+0.021<br>0.000	+0.020<br>0.000	+0.023<br>0.000	+0.028<br>0.000	+0.019<br>0.000	+0.020<br>0.000	+0.025<br>0.000	+0.019<br>0.009
**4d** At least one carbonate thermodynamically stable	801	+0.015<br>0.000	+0.018<br>0.000	+0.017<br>0.000	+0.021<br>0.000	+0.021<br>0.000	+0.021<br>0.000	+0.017<br>0.000	+0.018<br>0.000	+0.021<br>0.000		+0.019<br>0.000	+0.018<br>0.000	+0.022<br>0.000	+0.018<br>0.000	+0.018<br>0.000	+0.020<br>0.000	+0.018<br>0.000
**3b** Oxide supported	322	+0.014<br>0.000	+0.015<br>0.006	+0.016<br>0.000	+0.020<br>0.000	+0.019<br>0.001	+0.020<br>0.000	+0.016<br>0.000	+0.016<br>0.000	+0.020<br>0.000	+0.019<br>0.000		+0.016<br>0.001	+0.022<br>0.000	+0.017<br>0.000	+0.016<br>0.001	+0.018<br>0.000	+0.015<br>0.011
**4e** No thermodynamically stable carbonate	86	+0.014<br>0.000	+0.013<br>0.001	+0.015<br>0.000	+0.020<br>0.000	+0.022<br>0.000	+0.018<br>0.003	+0.015<br>0.000	+0.016<br>0.000	+0.023<br>0.000	+0.018<br>0.000	+0.016<br>0.001		+0.022<br>0.000	+0.015<br>0.000	+0.013<br>0.002	+0.016<br>0.001	+0.008<br>0.201
**4f** At least one carbonate thermodynamically stable	236	+0.016<br>0.000	+0.023<br>0.001	+0.017<br>0.000	+0.029<br>0.000	+0.030<br>0.000	+0.031<br>0.000	+0.017<br>0.000	+0.019<br>0.000	+0.028<br>0.000	+0.022<br>0.000	+0.022<br>0.000	+0.022<br>0.000		+0.022<br>0.000	+0.023<br>0.000	+0.029<br>0.000	+0.027<br>0.001
**2c** Self-supported	263	+0.014<br>0.000	+0.013<br>0.002	+0.015<br>0.000	+0.019<br>0.000	+0.018<br>0.000	+0.018<br>0.000	+0.015<br>0.000	+0.016<br>0.000	+0.019<br>0.000	+0.018<br>0.000	+0.017<br>0.000	+0.015<br>0.000	+0.022<br>0.000		+0.014<br>0.000	+0.016<br>0.000	+0.011<br>0.026
**4g** No thermodynamically stable carbonate	173	+0.014<br>0.000	+0.010<br>0.044	+0.015<br>0.000	+0.019<br>0.000	+0.019<br>0.001	+0.017<br>0.004	+0.015<br>0.000	+0.016<br>0.000	+0.020<br>0.000	+0.018<br>0.000	+0.016<br>0.001	+0.013<br>0.002	+0.023<br>0.000	+0.014<br>0.000		+0.015<br>0.003	+0.006<br>0.374
**4h** At least one carbonate thermodynamically stable	90	+0.014<br>0.000	+0.012<br>0.018	+0.016<br>0.000	+0.023<br>0.000	+0.025<br>0.000	+0.023<br>0.002	+0.016<br>0.000	+0.017<br>0.000	+0.025<br>0.000	+0.020<br>0.000	+0.018<br>0.000	+0.016<br>0.001	+0.029<br>0.000	+0.016<br>0.000	+0.015<br>0.003		+0.007<br>0.360
**2d** Not assigned	47	+0.013<br>0.000	-0.004<br>0.584	+0.015<br>0.000	+0.017<br>0.023	+0.014<br>0.158	+0.014<br>0.178	+0.014<br>0.000	+0.015<br>0.000	+0.019<br>0.009	+0.018<br>0.000	+0.015<br>0.011	+0.008<br>0.201	+0.027<br>0.001	+0.011<br>0.026	+0.006<br>0.374	+0.007<br>0.360	

Supplementary Table 11b Matrix of β_T regression coefficients. Each cell gives the β_T regression coefficient and p-value between a pair of property groups.

Supplementary Note 11c Final model: Matrix of $\beta_{CH4/O2}$ regression coefficients and their p-values

Each multivariate regression analysis with p_{CH4}/p_{O2} compensation produces also a matrix of $\beta_{CH4/O2}$ values and the corresponding p values. The matrix resulting for the final model is provided below. The large majority of regression tests indicate a statistically significant influence of decreasing C_2 yield for increased p_{CH4}.

Group	n	0 All catalysts	1a No carb	1b Can form a carbonate	2a Unsupp.	4a	4b	2b Supported	3a Carb supp.	4c	4d	3b Oxide supp.	4e	4f	2c Self-supp.	4g	4h	2d n.a.
**0** All catalysts	1802		-0.44<br>0.000	-0.46<br>0.000	-0.43<br>0.000	-0.44<br>0.000	-0.45<br>0.000	-0.47<br>0.000	-0.47<br>0.000	-0.45<br>0.000	-0.47<br>0.000	-0.48<br>0.000	-0.43<br>0.000	-0.50<br>0.000	-0.44<br>0.000	-0.45<br>0.000	-0.45<br>0.000	-0.46<br>0.000
**1a** Cannot a form carbonate	38	-0.44<br>0.000		-0.45<br>0.000	-0.29<br>0.000	-0.15<br>0.029	-0.30<br>0.000	-0.47<br>0.000	-0.45<br>0.000	-0.15<br>0.075	-0.48<br>0.000	-0.52<br>0.000	-0.07<br>0.295	-0.73<br>0.000	-0.23<br>0.000	-0.24<br>0.001	-0.13<br>0.126	-0.11<br>0.337
**1b** Can form a carbonate	1764	-0.46<br>0.000	-0.45<br>0.000		-0.44<br>0.000	-0.44<br>0.000	-0.46<br>0.000	-0.48<br>0.000	-0.47<br>0.000	-0.46<br>0.000	-0.48<br>0.000	-0.49<br>0.000	-0.44<br>0.000	-0.51<br>0.000	-0.45<br>0.000	-0.46<br>0.000	-0.46<br>0.000	-0.46<br>0.000
**2a** Unsupported	197	-0.43<br>0.000	-0.29<br>0.000	-0.44<br>0.000		-0.31<br>0.000	-0.38<br>0.000	-0.45<br>0.000	-0.43<br>0.000	-0.33<br>0.000	-0.45<br>0.000	-0.45<br>0.000	-0.27<br>0.000	-0.52<br>0.000	-0.32<br>0.000	-0.34<br>0.000	-0.33<br>0.000	-0.33<br>0.000
**4a** No thermodynamically stable carbonate	57	-0.44<br>0.000	-0.15<br>0.029	-0.44<br>0.000	-0.31<br>0.000		-0.34<br>0.000	-0.46<br>0.000	-0.44<br>0.000	-0.21<br>0.002	-0.45<br>0.000	-0.46<br>0.000	-0.15<br>0.012	-0.56<br>0.000	-0.26<br>0.000	-0.27<br>0.000	-0.23<br>0.001	-0.24<br>0.011
**4b** At least one carbonate thermodynamically stable	140	-0.45<br>0.000	-0.30<br>0.000	-0.46<br>0.000	-0.38<br>0.000	-0.34<br>0.000		-0.47<br>0.000	-0.46<br>0.000	-0.36<br>0.000	-0.48<br>0.000	-0.52<br>0.000	-0.28<br>0.000	-0.65<br>0.000	-0.35<br>0.000	-0.37<br>0.000	-0.36<br>0.000	-0.37<br>0.000
**2b** Supported	1257	-0.47<br>0.000	-0.47<br>0.000	-0.48<br>0.000	-0.45<br>0.000	-0.46<br>0.000	-0.47<br>0.000		-0.49<br>0.000	-0.47<br>0.000	-0.50<br>0.000	-0.52<br>0.000	-0.45<br>0.000	-0.55<br>0.000	-0.46<br>0.000	-0.48<br>0.000	-0.47<br>0.000	-0.49<br>0.000
**3a** Carbonate supported	935	-0.47<br>0.000	-0.45<br>0.000	-0.47<br>0.000	-0.43<br>0.000	-0.44<br>0.000	-0.46<br>0.000	-0.49<br>0.000		-0.45<br>0.000	-0.50<br>0.000	-0.52<br>0.000	-0.43<br>0.000	-0.55<br>0.000	-0.45<br>0.000	-0.47<br>0.000	-0.46<br>0.000	-0.48<br>0.000
**4c** No thermodynamically stable carbonate	134	-0.45<br>0.000	-0.15<br>0.075	-0.46<br>0.000	-0.33<br>0.000	-0.21<br>0.002	-0.36<br>0.000	-0.47<br>0.000	-0.45<br>0.000		-0.48<br>0.000	-0.51<br>0.000	-0.16<br>0.024	-0.66<br>0.000	-0.29<br>0.000	-0.31<br>0.000	-0.25<br>0.002	-0.30<br>0.003
**4d** At least one carbonate thermodynamically stable	801	-0.47<br>0.000	-0.48<br>0.000	-0.48<br>0.000	-0.45<br>0.000	-0.45<br>0.000	-0.48<br>0.000	-0.50<br>0.000	-0.50<br>0.000	-0.48<br>0.000		-0.54<br>0.000	-0.45<br>0.000	-0.59<br>0.000	-0.47<br>0.000	-0.49<br>0.000	-0.48<br>0.000	-0.51<br>0.000
**3b** Oxide supported	322	-0.48<br>0.000	-0.52<br>0.000	-0.49<br>0.000	-0.45<br>0.000	-0.46<br>0.000	-0.52<br>0.000	-0.52<br>0.000	-0.52<br>0.000	-0.51<br>0.000	-0.54<br>0.000		-0.45<br>0.000	-0.76<br>0.000	-0.48<br>0.000	-0.54<br>0.000	-0.53<br>0.000	-0.60<br>0.000
**4e** No thermodynamically stable carbonate	86	-0.43<br>0.000	-0.07<br>0.295	-0.44<br>0.000	-0.27<br>0.000	-0.15<br>0.012	-0.28<br>0.000	-0.45<br>0.000	-0.43<br>0.000	-0.16<br>0.024	-0.45<br>0.000	-0.45<br>0.000		-0.55<br>0.000	-0.22<br>0.000	-0.23<br>0.001	-0.15<br>0.028	-0.17<br>0.056
**4f** At least one carbonate thermodynamically stable	236	-0.50<br>0.000	-0.73<br>0.000	-0.51<br>0.000	-0.52<br>0.000	-0.56<br>0.000	-0.65<br>0.000	-0.55<br>0.000	-0.55<br>0.000	-0.66<br>0.000	-0.59<br>0.000	-0.76<br>0.000	-0.55<br>0.000		-0.58<br>0.000	-0.70<br>0.000	-0.73<br>0.000	-0.91<br>0.000
**2c** Self-supported	263	-0.44<br>0.000	-0.23<br>0.000	-0.45<br>0.000	-0.32<br>0.000	-0.26<br>0.000	-0.35<br>0.000	-0.46<br>0.000	-0.45<br>0.000	-0.29<br>0.000	-0.47<br>0.000	-0.48<br>0.000	-0.22<br>0.000	-0.58<br>0.000		-0.32<br>0.000	-0.28<br>0.000	-0.31<br>0.000
**4g** No thermodynamically stable carbonate	173	-0.45<br>0.000	-0.24<br>0.001	-0.46<br>0.000	-0.34<br>0.000	-0.27<br>0.000	-0.37<br>0.000	-0.48<br>0.000	-0.47<br>0.000	-0.31<br>0.000	-0.49<br>0.000	-0.54<br>0.000	-0.23<br>0.001	-0.70<br>0.000	-0.32<br>0.000		-0.30<br>0.000	-0.34<br>0.000
**4h** At least one carbonate thermodynamically stable	90	-0.45<br>0.000	-0.13<br>0.126	-0.46<br>0.000	-0.33<br>0.000	-0.23<br>0.001	-0.36<br>0.000	-0.47<br>0.000	-0.46<br>0.000	-0.25<br>0.002	-0.48<br>0.000	-0.53<br>0.000	-0.15<br>0.028	-0.73<br>0.000	-0.28<br>0.000	-0.30<br>0.000		-0.24<br>0.035
**2d** Not assigned	47	-0.46<br>0.000	-0.11<br>0.337	-0.46<br>0.000	-0.33<br>0.000	-0.24<br>0.011	-0.37<br>0.000	-0.49<br>0.000	-0.48<br>0.000	-0.30<br>0.003	-0.51<br>0.000	-0.60<br>0.000	-0.17<br>0.056	-0.91<br>0.000	-0.31<br>0.000	-0.34<br>0.000	-0.24<br>0.035	

Supplementary Table 11c Matrix of $\beta_{CH4/O2}$ regression coefficients. Each cell gives the $\beta_{CH4/O2}$ regression coefficient and p-value between a pair of property groups.

Supplementary Note 12 Generation of density plots of Y_{C2} via Epanechnikov kernel density function

The proposed method applies decision rules in order to sort all reported catalysts into groups of similar physico-chemical properties. The dataset reports for each catalyst i also an indicator of OCM performance, i.e. $Y_{C2,\,i}$. The OCM performance of the resulting property groups can be evaluated based on the distribution of $Y_{C2,i}$ values within each group as well as the deduced group-averaged $\bar{Y}_{C2}$. So-called density plots of C_2 yield were generated via Epanechnikov kernel density function in order to visualize the Y_{C2} distribution for each group (see e.g. Y_{C2} density plots in Fig. 4 of the main article).

A kernel density function describes a nonparametric method, which is used to smooth random data variability and to illustrate systematic variability at the same time[23]. The basis of kernel densities is the principle of local averaging. To calculate a local average value on a certain value of estimation x, not only observations featuring exactly the value x are considered in the calculation, but also the surrounding observations with values between $x-h$ and $x+h$, where h is called the bandwidth. Observations (y_i, x_i) close to the value of estimation x typically get larger weights for calculation of a weighted average[23]. Variable u typifies the distance between a considered observation x_i and the value of estimation x divided by the bandwidth h.

$$u = \frac{x - x_i}{h} \qquad (1)$$

Equation (2) shows how the kernel weight $K(u)$ is calculated using the Epanechnikov kernel density function[24]. $K(u)$ indicates whether and to what extent an observation x_i is considered in the calculation of the density at the value of estimation x.

$$K(u) = \begin{cases} \frac{3}{4}(1-u^2)\, for\ \{|u| \le 1\} \\ 0\, for\ \{|u| > 1\} \end{cases} \qquad (2)$$

Observations featuring exactly the point of estimation x get the largest weight of 0.75. The Supplementary Figure 12 illustrates the shape of the Epanechnikov kernel density. The Epanechnikov kernel density considers both a weight and a cutoff limit.

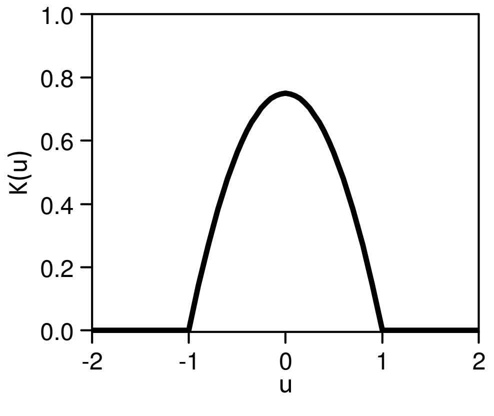

Supplementary Figure 12 Illustration of the shape of the Epanechnikov kernel density.

Supplementary Note 13 Hypothesis 4: Frequent cation combinations, yield densities and regression results

Hypothesis 4 compares for the subgroups 2a, 2c, 3a and 3c the decomposition temperature of a carbonate with the actual OCM test temperature as reported in the dataset. A catalyst is assigned to a respective subgroup "carbonate thermodynamically stable" if the OCM reaction was measured at a temperature that is lower than the actual temperature at which the most stable contained carbonate starts to decompose, corrected for an offset that accounts for experimental uncertainties and dependence of carbonate formation equilibria on CO_2 partial pressure (here: $T_{OCM\ measurement} < T_{carbonate\ decomposition}$ + 100K; for other offset values see Supplementary Note 14 h-k). In the groups formed by hypothesis 4, the role of the thermodynamically most stable carbonate is often taken on by an alkaline, alkaline earth or lanthanide elements. Such elements are typically absent in catalysts that do not fulfil the criterion of a thermodynamically stable carbonate.

Group **4a ("Unsupported → No thermodynamically stable carbonate")** comprises a relatively small number of catalysts (N = 57). The most common cation combinations contain heavy metals (i.e. Pb, Bi) which form carbonates with low decomposition temperatures. In the group **4b ("Unsupported → At least one carbonate thermodynamically stable")**, heavy metals are typically combined with alkaline metals, which form carbonates with relatively high decomposition temperatures.

Group **4c ("Carbonate supported → No thermodynamically stable carbonate")** comprises mostly binary combinations of Mg or Ca with Pb or La. In the group **4d ("Carbonate supported → At least one carbonate thermodynamically stable")** mainly binary combinations of an alkaline and an alkaline earth element (i.e. Li-Mg, Na-Ca, Li-Ca) and La containing catalysts (i.e. La-Sr, La-Ba) occur. Note that Ca and La have intermediate carbonate decomposition temperatures. This makes their classification very sensitive towards the measurement temperature, so that these elements appear in both subgroups.

Group **4e ("Oxide supported → No thermodynamically stable carbonate")** typically contains cation compositions that are binary combinations of Pb or transition metals (i.e. Mn, Co) supported on Al or Si. In contrast, the group **4f ("Oxide supported → At least one carbonate thermodynamically stable")** is dominated by variations of the Na-Mn-W-Si system, in which Na is able to form a carbonate under OCM conditions. Additionally, binary combinations of alkaline metals or alkaline earth metals and Al, Si, or Ti are located in this group.

The subgroups **4g and 4h of the "Self-supported" group** represent catalysts that contain only one cation. Common elements unable to form a stable carbonate under their respective OCM conditions are Mg, Ca and lanthanides (4g). Frequently reported elements with the ability form a thermodynamically stable carbonate under OCM conditions (4h) are Ca, Sr, Ba and La. Note that again Ca and La often appear in both subgroups, depending on the employed / reported OCM measurement temperature.

The regression results indicate that the catalyst property "Carbonate thermodynamically stable" has a clearly beneficial influence on OCM performance. For the support property group **2a ("Unsupported", subgroups 4a vs. 4b)**, the regression coefficient amounts to $\beta_{group} = +2.04$ with sufficient statistical significance. Also for the groups **3a ("Carbonate supported", 4c vs. 4d)** and **3b ("Oxide supported", 4e vs. 4f)** the respective values are $\beta_{group} = +2.36$ and $\beta_{group} = +5.11$ are high and statistically significant. Hence, the positive effect is confirmed for three different types of catalyst groups. Only for group **2c ("Self-supported", 4g vs. 4h)** no statistically significant result was obtained. This might be related to the fact that all catalyst in group 2c contained only one cation, and can therefore not provide the "support function" and the "carbonate function" via two different cations.

Supplementary Note 14 Robustness test: Variation of descriptor rule parameters for definition of property groups

An essential criterion in the evaluation of model quality is the robustness of its findings against the modifications of model parameters and regression variables. The robustness of the final model (Figure 3, Figure 4) was therefore assessed via systematic variation of the parameter values employed by the sorting rules (4) (Figure 2). The varied parameters include (i) the minimum weight fraction required for an oxide to be considered as support (level 2), (ii) the factor used to calculate the Tammann temperature from the melting point of the most stable oxide (level 2), and (iii) the offset between the reported OCM temperature and the decomposition temperature of the most stable carbonate formed by a catalyst (level 4). Moreover, the regression was performed (iv) without correction for temperature and pressure ratio p_{CH4}/p_{O2} ($\beta_T \overset{!}{=} \beta_{CH4/O2} \overset{!}{=} 0$), (v) with weights that compensate for the different observation frequencies of catalysts of the same nominal cation-combination, (vi) with $\ln(Y_{C2})$ as independent variable instead of Y_{C2}, and (vii) with a so-called "robust regression" approach (see supplement for details: (i) Supplementary Note 14a-d; (ii) Supplementary Note 14e-f; (iii) Supplementary Note 14j-k; (iv) Supplementary Note 15; (v) Supplementary Note 16; (vi) Supplementary Note 17; (vii) Supplementary Note 18).

In our approach, the properties of a catalyst are described by descriptor rules and sorting rules that evaluate the physico-chemical properties of elements contained in a catalyst. Some of the descriptors are defined by numerical parameters (see e.g. Supplementary Note 6). A variation of the respective parameter values can change to which property group a catalyst will be assigned. To test for robustness, we varied the following descriptor parameters individually, while keeping all other parameters constant:

Weight fraction parameter:	30%, 40%, **50%**, 60%, 70%	(Supplementary Note 14a-d)
Tammann temperature factor:	0.40, 0.50, **0.60**, 0.70	(Supplementary Note 14e-g)
Temperature offset for carbonate stability:	0 K, 50 K, **100 K**, 150 K, 200 K	(Supplementary Note 14h-k)

Values that correspond to the final model (main article, Fig. 4) are marked in **bold**. The changes in property group compositions resulting from the parameter variation are discussed qualitatively below. The subsequently presented Supplementary Figures 14a-k provide the corresponding detailed regression results.

The vast majority of the main effects on Y_{C2} (approximate effect size, sign) identified by the final model (Fig. 4) was preserved throughout the parameter variations (Supplementary Figure 14a-k). Only in few cases, some of the identified effects became statistically insignificant (e.g. 4a/4b in Supplementary Figure 14a, b, h, i). Moreover, most catalysts remained in their respective property group throughout the parameter variations. Hence, the final model proves to be very robust towards variation of the tested descriptor parameters.

A variation of the weight fraction parameter in the support descriptor rule (equation (1) in Supplementary Note 6) mainly shift the position of catalysts between the groups “Unsupported” and “Supported” in hypothesis 2. Increasing the threshold corresponds to less catalyst components that fulfil the criterion, so that the respective catalysts move into the group “Unsupported” (2a) and vice versa. This effect occurs more pronounced when the component that is assumed to provide the support function possesses a low molar mass (i.e. Al, Mg) compared to components with higher molar mass (i.e. Pb, Bi). Values below 50% weight fraction allow more than one component of a catalyst to fulfil the support function, which can cause ambiguity in the third hypothesis.

A variation of the Tammann temperature factor (equation (2) in Supplementary Note 6) in the support descriptor rule mostly affected catalysts containing SiO_2 as a support. SiO_2-based catalysts measured at 800 °C fulfil the support criterion only when the Tammann factor exceed 0.54. However, increasing the factor further above 0.6 had very little influence on the assignment of the support function.

A variation of the temperature offset that evaluates the thermodynamic carbonate stability in hypothesis 4 (equation (3) in Supplementary Note 6) influences the group assignment of catalysts that containing specific elements as their most stable carbonates. For example, decreasing the offset below 100 K causes Li_2CO_3 to be considered as thermodynamically unstable for many catalysts. However, experimental studies indicate that Li_2CO_3 is often present on such catalysts after OCM testing[25-27]. Increasing the offset value to 150 K causes mainly $CaCO_3$ and $La_2O_2CO_3$ to be considered as thermodynamically stable. However, at some point the subgroups “No thermodynamically stable carbonate” contain only few remaining catalysts, which reduces the statistical value of a respective group comparison.

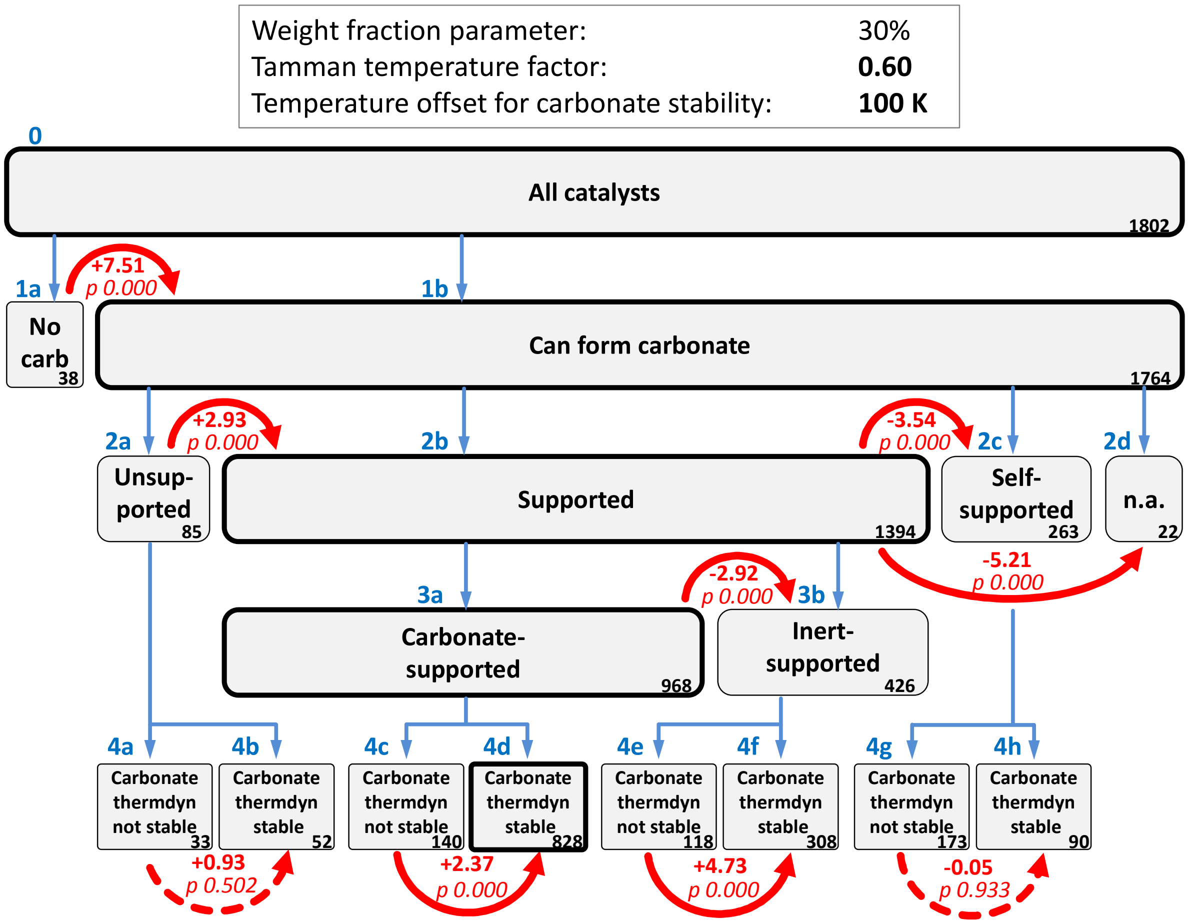

Supplementary Figure 14a Variation of wt% descriptor parameter to 30% and resulting catalyst property tree with hypotheses results. Reference numbers assigned to each property group are shown in blue. The number of catalysts (N) assigned to each property group is provided in the bottom right corner of each box and indicated for hypothesis 1, 2 and 3 also by the corresponding box width. Red arrows indicate the direction of each hypotheses that compares two property groups via regression analysis and are labeled with the obtained β_{group} regression coefficient (~ $\Delta\bar{Y}_{C2}$) and p-value. The path towards the group with the highest OCM performance is marked by boxes with bold fringe lines.

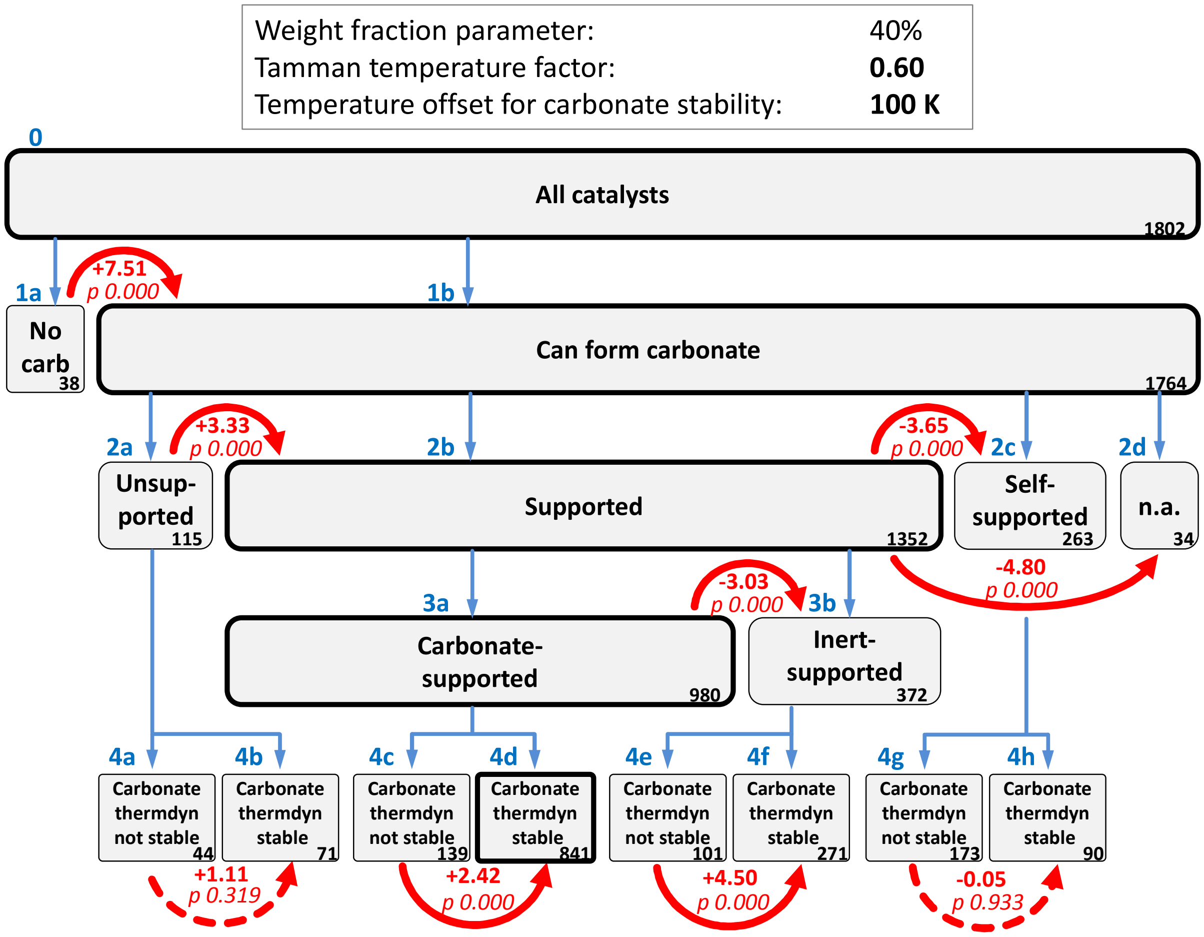

Supplementary Figure 14b Variation of wt% descriptor parameter to 40% and resulting catalyst property tree with hypotheses results.

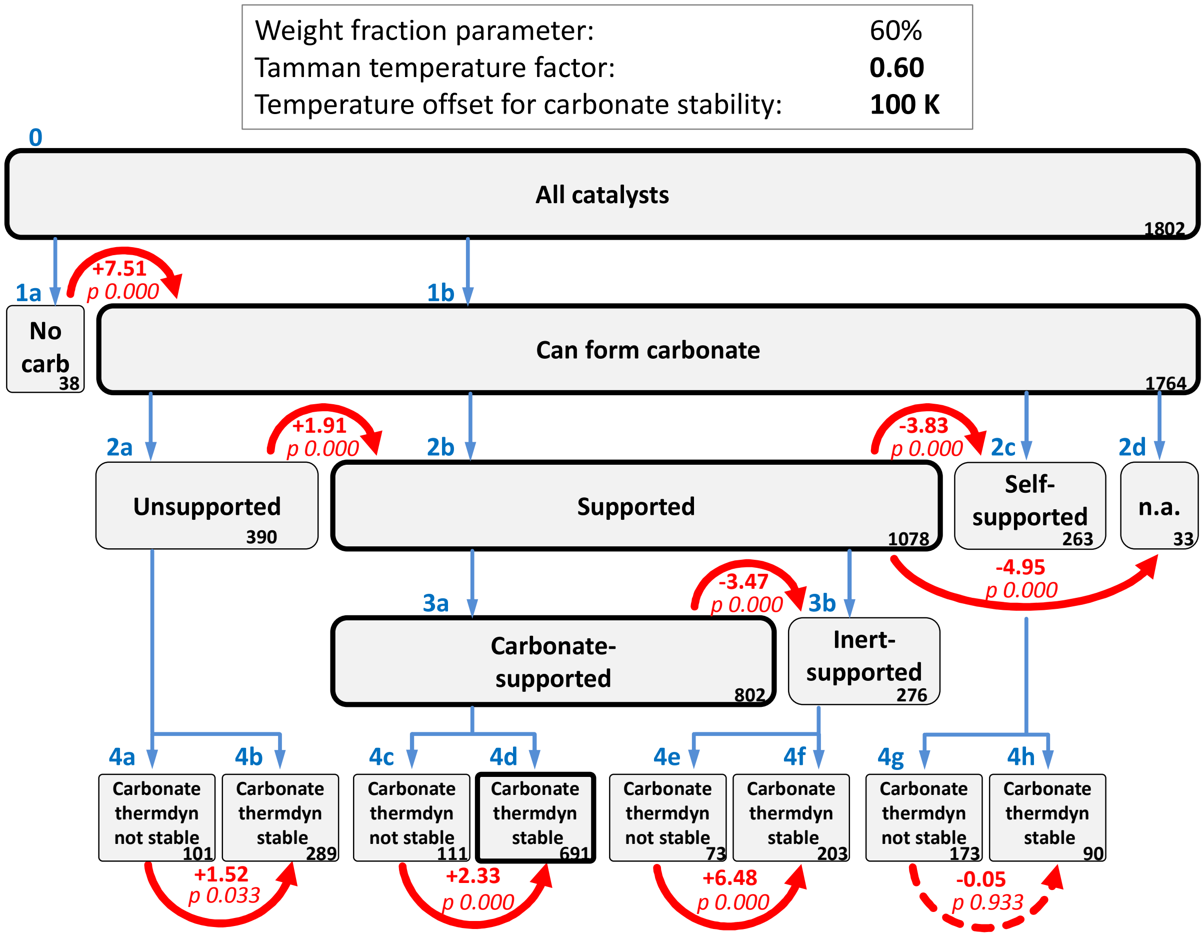

Supplementary Figure 14c Variation of wt% descriptor parameter to 60% and resulting catalyst property tree with hypotheses results.

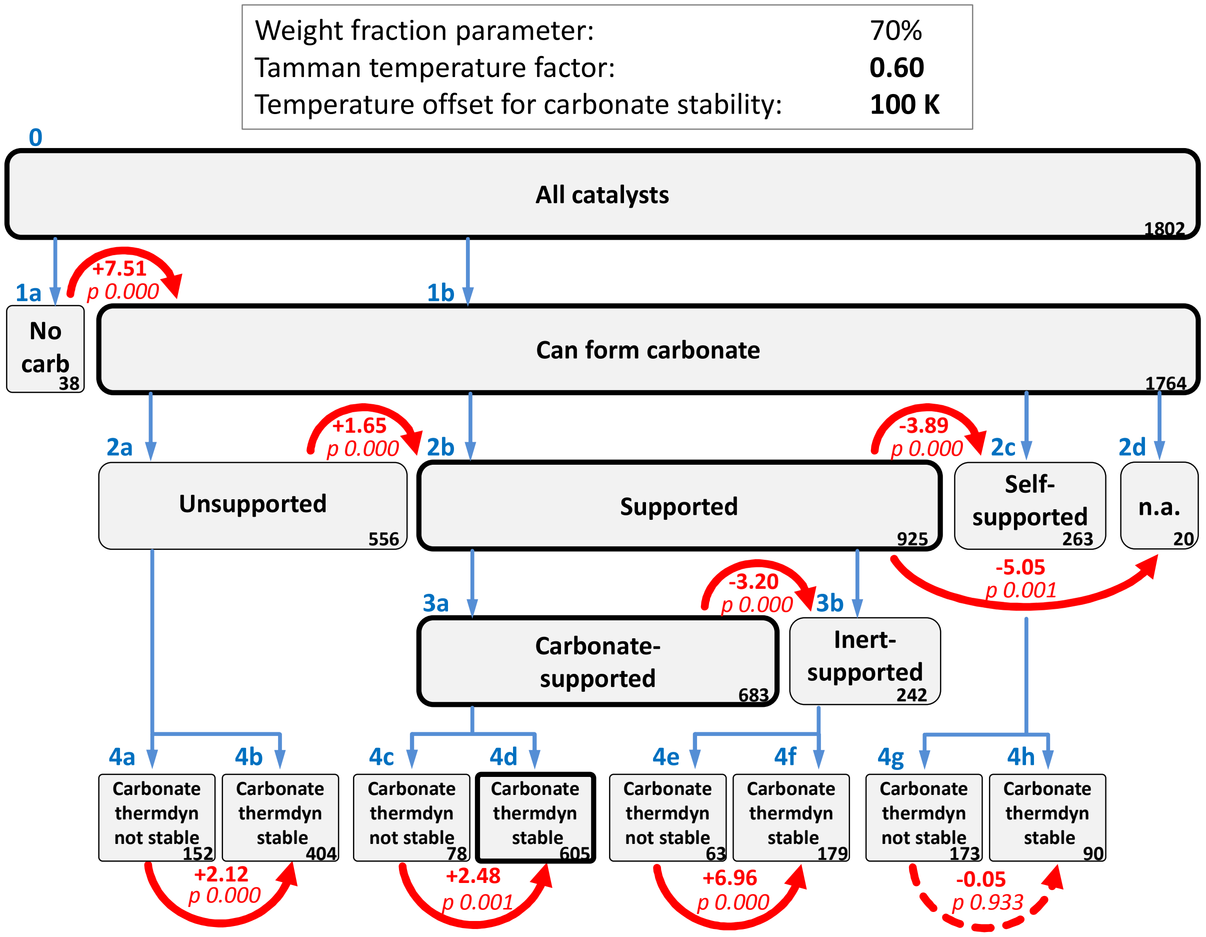

Supplementary Figure 14d Variation of wt% descriptor parameter to 70% and resulting catalyst property tree with hypotheses results.

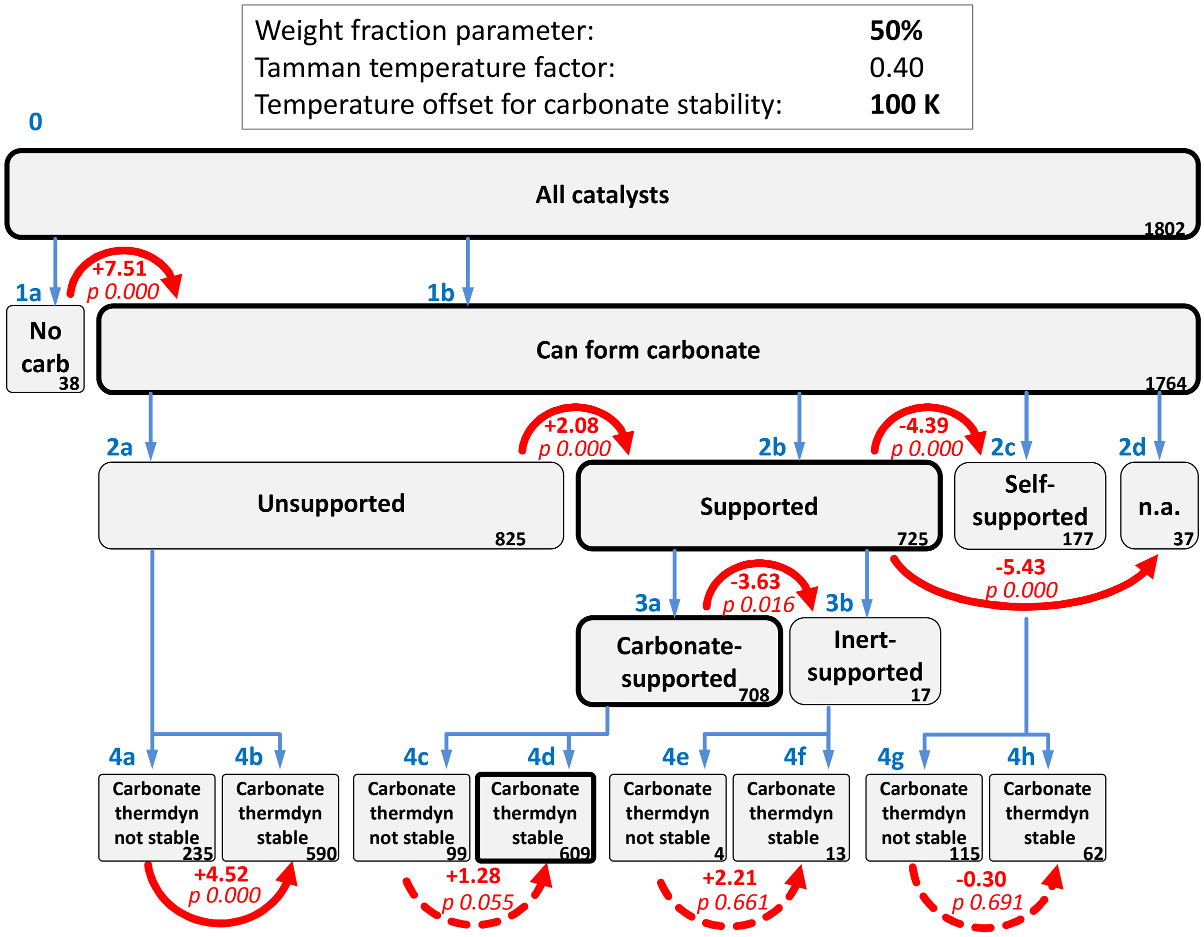

Supplementary Figure 14e Variation of Tammann factor descriptor parameter to 0.40 and resulting catalyst property tree with hypotheses results.

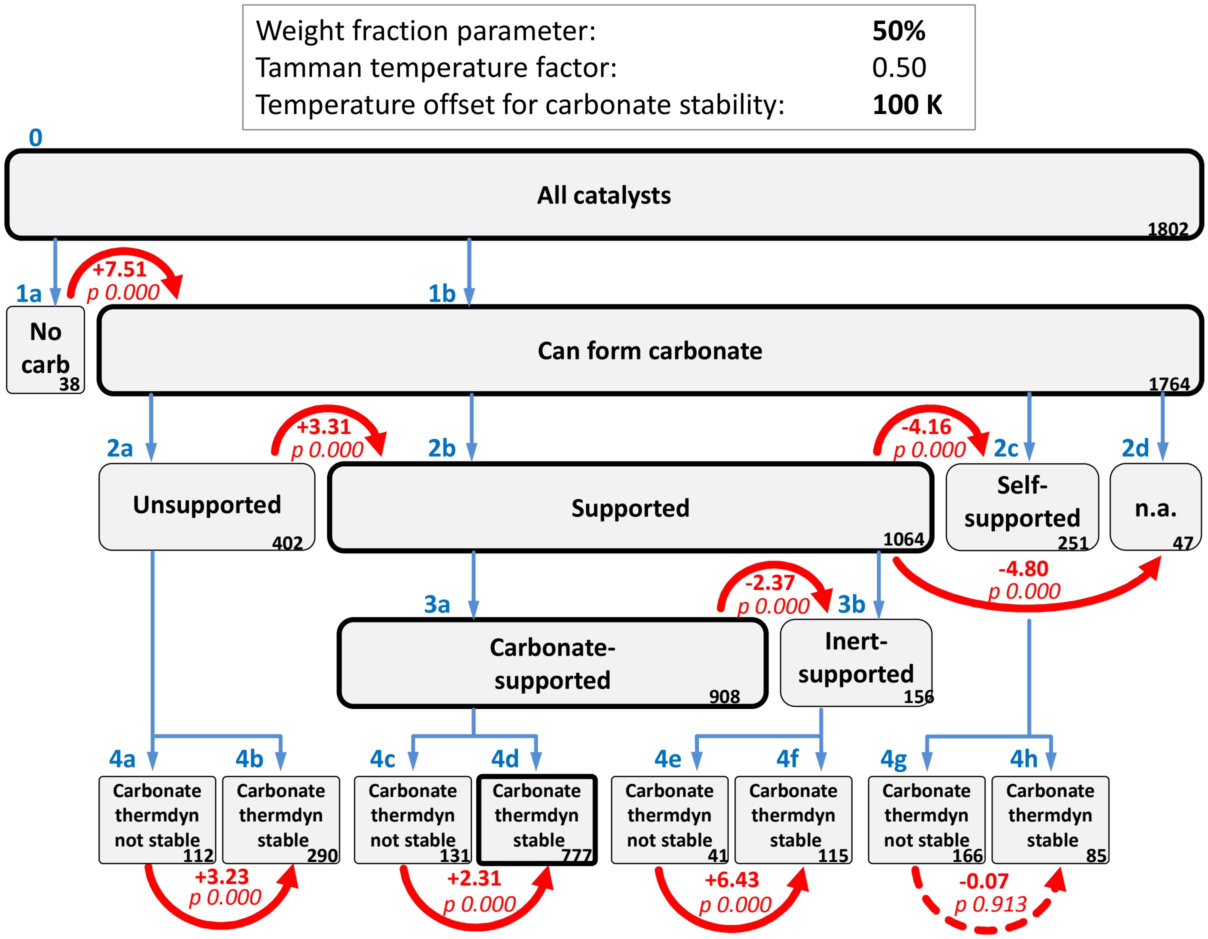

Supplementary Figure 14f Variation of Tammann factor descriptor parameter to 0.50 and resulting catalyst property tree with hypotheses results.

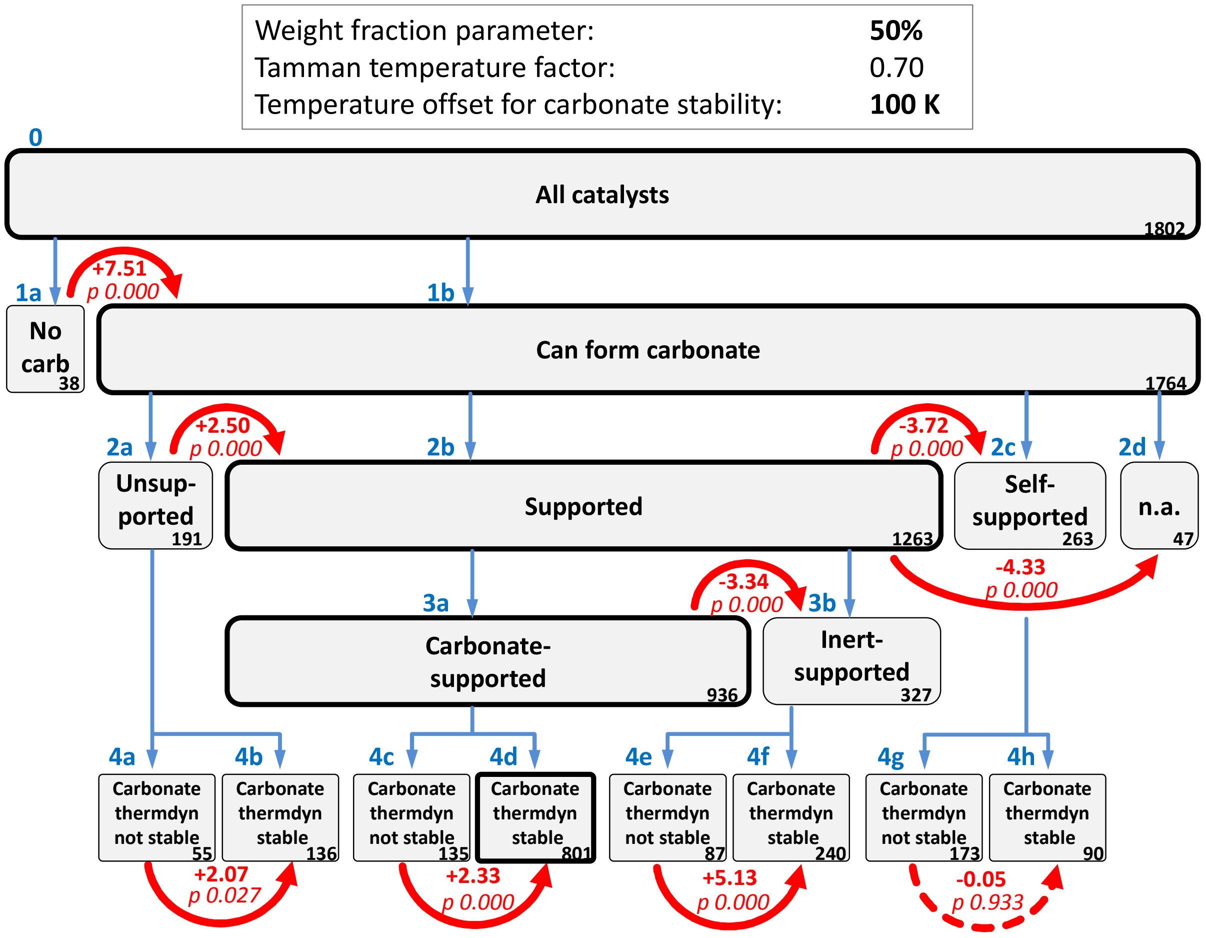

Supplementary Figure 14g Variation of Tammann factor descriptor parameter to 0.70 and resulting catalyst property tree with hypotheses results.

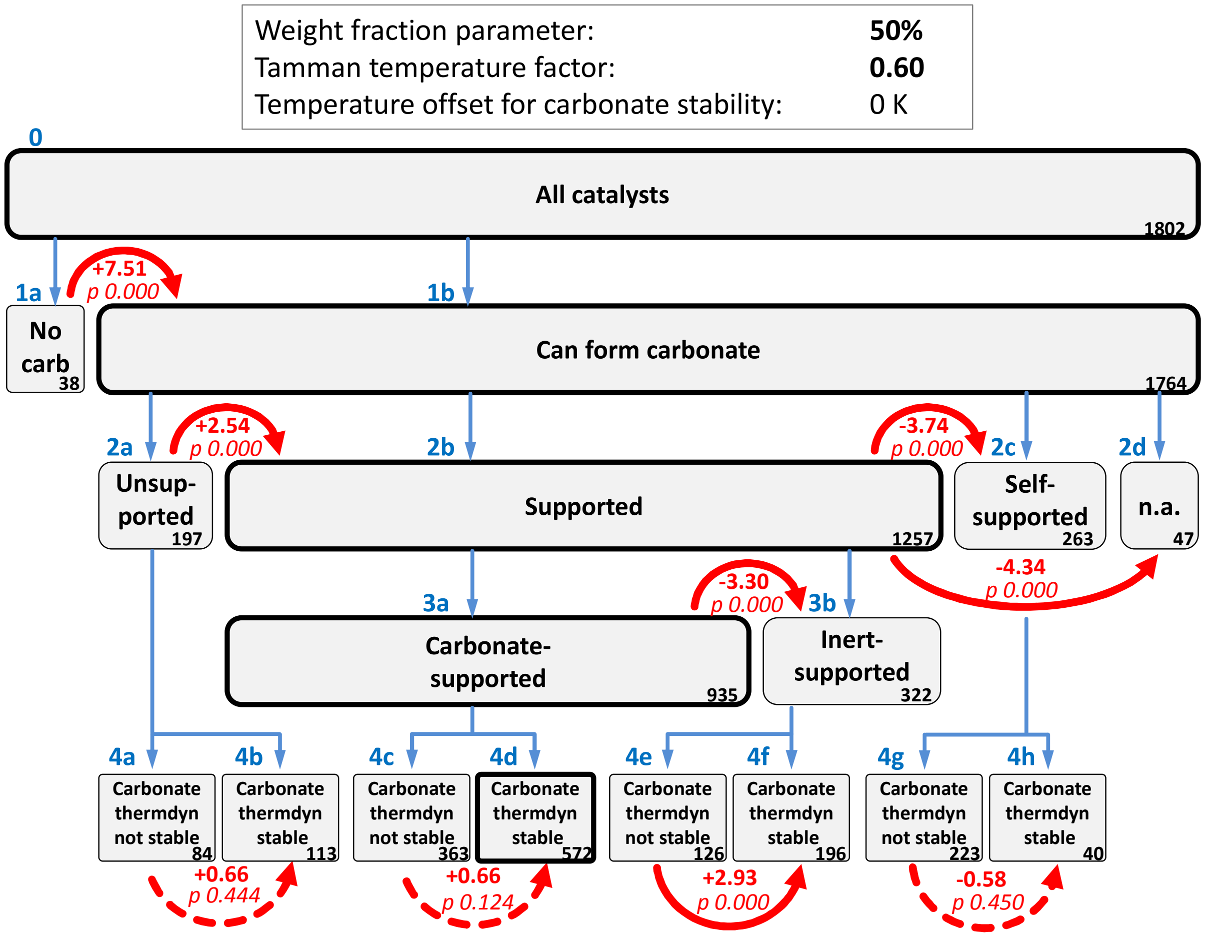

Supplementary Figure 14h Variation of carbonate stability offset descriptor parameter to 0 K and resulting catalyst property tree with hypotheses results.

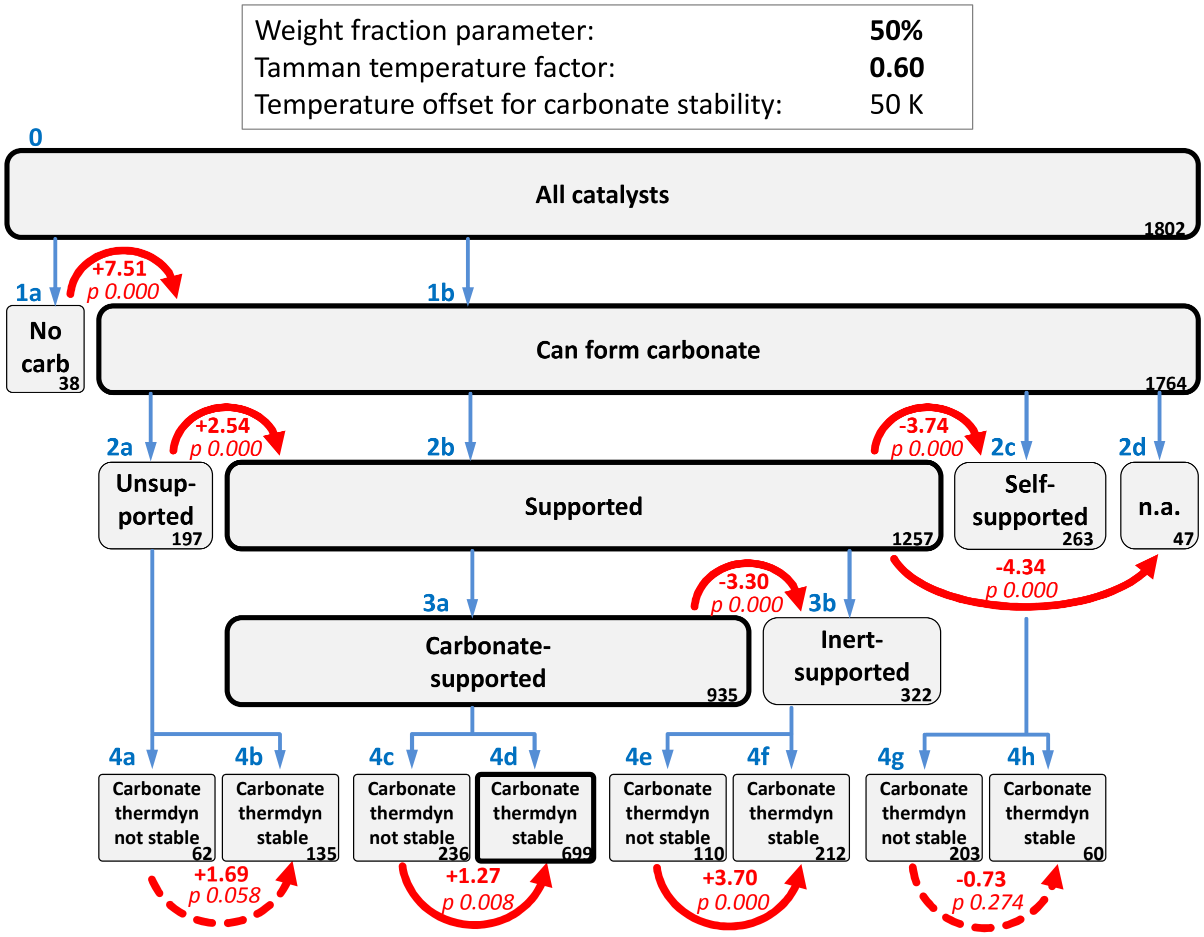

Supplementary Figure 14i Variation of carbonate stability offset descriptor parameter to 50 K and resulting catalyst property tree with hypotheses results.

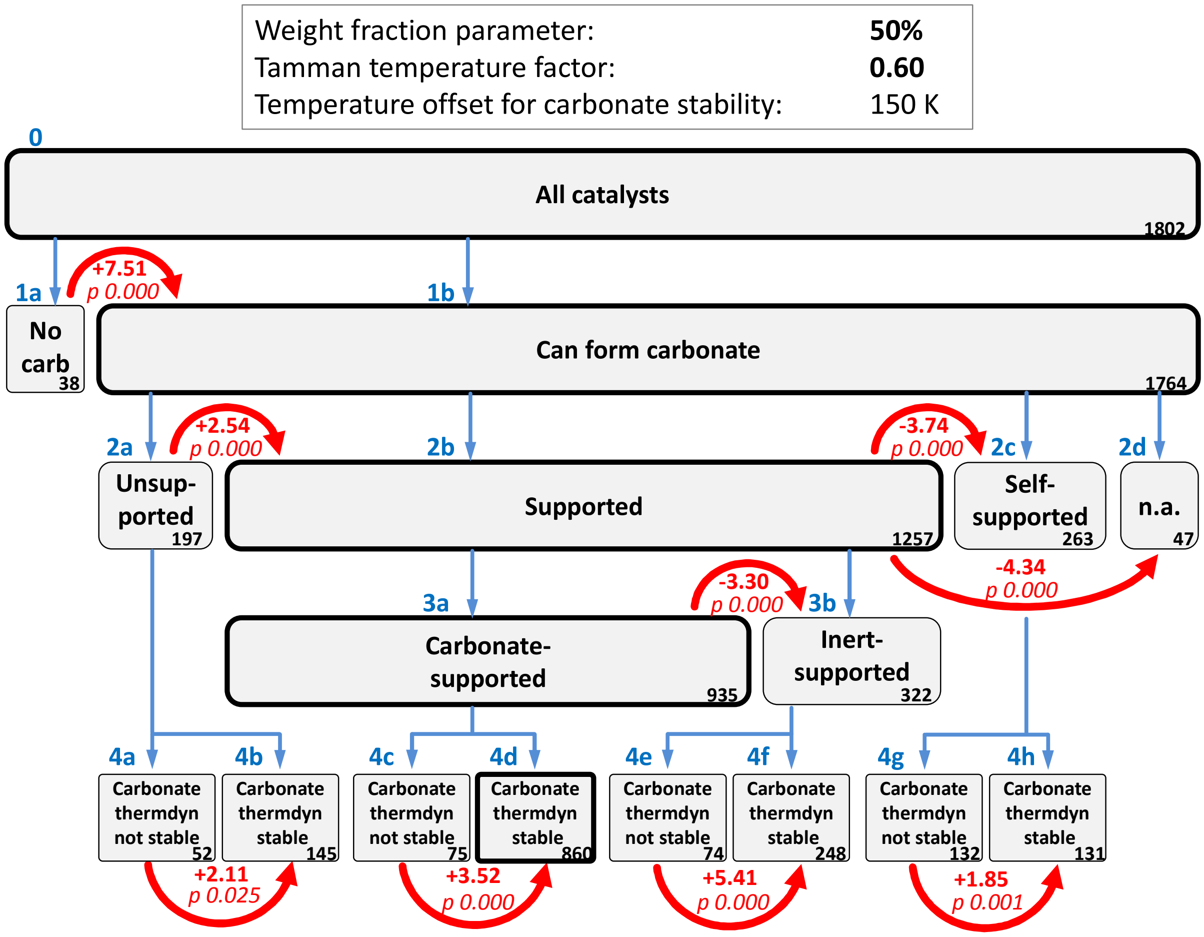

Supplementary Figure 14j Variation of carbonate stability offset descriptor parameter to 150 K and resulting catalyst property tree with hypotheses results.

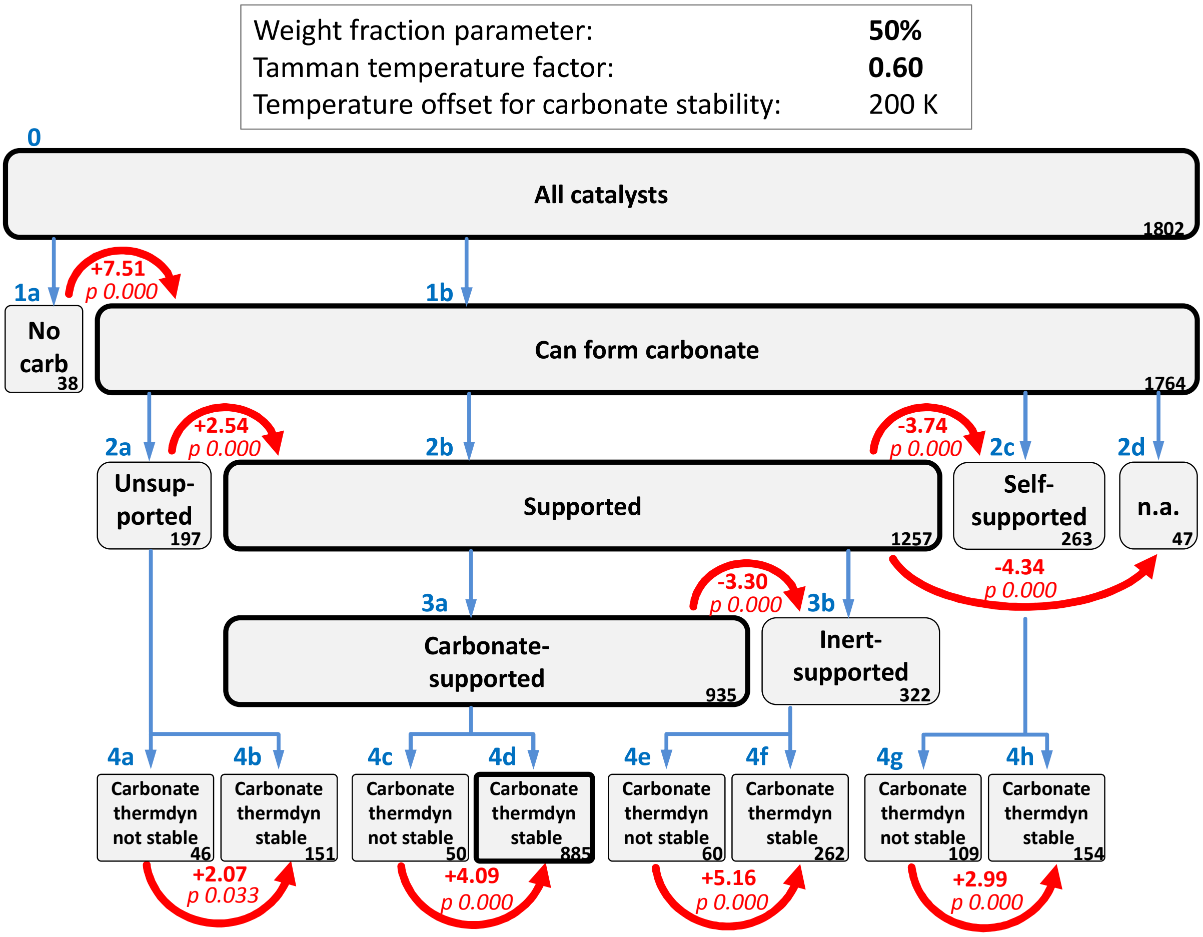

Supplementary Figure 14k Variation of carbonate stability offset descriptor parameter to 200 K and resulting catalyst property tree with hypotheses results.

Supplementary Note 15 Robustness test: Regression without compensation for reaction conditions

The reaction conditions temperature and CH_4/O_2 ratio strongly influence the experimentally observed C_2 yield of the OCM reaction as discussed in Supplementary Note 9. All the statistical analysis discussed so far (final model Fig. 4; Supplementary Note 14) compensate for variations in T and p_{CH4}/p_{O2} using a linear approximation for both variables (eq. 1). To test the models robustness the complete regression analysis was performed also without compensation for T and p_{CH4}/p_{O2} (eq. 2). Hence, regression coefficient β_{group} that indicates if a catalyst belongs to a tested catalyst property group remains as the only independent variable.

$$Y_i = \beta_0 + \beta_{group} \times D_{group,i} + \beta_T \times \Delta T_i + \beta_{CH4/O2} \times \Delta p_{CH4/O2,i} + U_i \quad (1)$$

$$Y_i = \beta_0 + \beta_{group} \times D_{group,i} + U_i \quad (2)$$

The obtained regression results are shown in Supplementary Figure 15. Note that the computed β_{group} regression coefficient equal now the difference of the mean value of the observed C_2 yields. The results indicate only minor differences when compared to the final model (Fig. 4) that employed a linear T and p_{CH4}/p_{O2} compensation. All main effects are preserved and remain statistically significant. Moreover, $\bar{Y}_{C2}$ computed for each terminal groups changes by less than 0.7 percentage points when compared to the final model (Fig. 4), providing further evidence for the excellent robustness of the developed model.

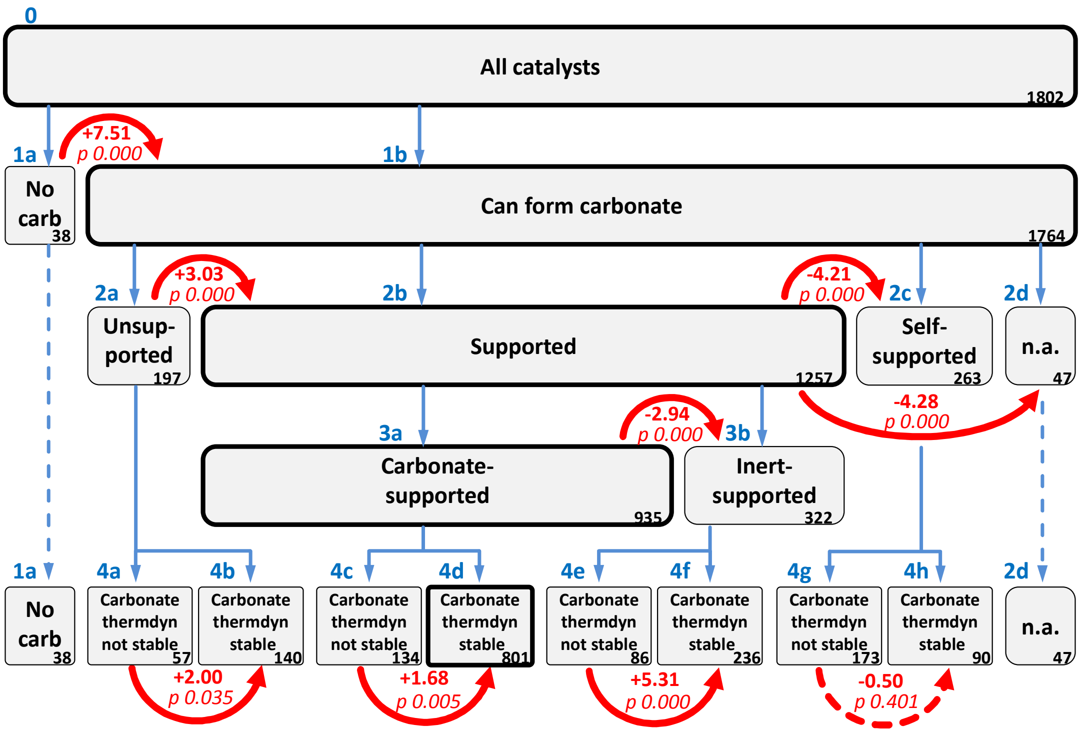

$\bar{Y}_{C2,\ uncorrected}$	2.11	6.28	8.28	10.05	11.73	4.65	9.97	6.70	6.20	6.48
β_{group} to 4d	+9.62	+4.45	+3.45	+1.68	-	+7.08	+1.76	+5.03	+5.53	+5.25
p-value	*0.000*	*0.000*	*0.000*	*0.005*	-	*0.000*	*0.001*	*0.000*	*0.000*	*0.000*

Supplementary Figure 15 Catalyst property tree with hypotheses results when the regression is performed without compensation for differences in linear T and p_{CH4}/p_{O2}.

Supplementary Note 16 Robustness test: Regression with weighting of similar catalyst compositions

In the dataset, some cation combinations are reported more frequently than others (i.e. Li-Mg, Na-Ca, see Supplementary Note 10). All the statistical analysis discussed so far (final model Fig. 4; Supplementary Note 14, Supplementary Note 15) weighted each observation equally. Hence, the frequently reported cation combinations have a stronger impact on the regression results. However, a robust model should provide similar results also when each cation combination has a similar impact on the regression analysis. Hence, the regression was also performed in a way that attached weights (the inverse number of catalysts of similar cation combination in the respective property group) to the squared residual U_i of each Y_{C2} observation. If e.g. the cation combination “Li-Mg” appeared 72 times in the group “Can form a carbonate”, the a factor of $\frac{1}{72}$ was attached to each observation of the cation combination “Li-Mg”.

The obtained regression results are presented in Supplementary Figure 16. The results indicate only minor differences when compared to the final model (Fig. 4) that did not include the respective weight factors. All main effects are preserved and remain statistically significant. The strongest change in the effect value $\Delta\bar{Y}_{C2}$ is observed for group 1a/1b (1.2 percentage points). Moreover, $\bar{Y}_{C2}$ computed for each terminal group changes also by less than 1.2 percentage point when compared to the final model (Fig. 4), which confirms the excellent robustness of the developed model.

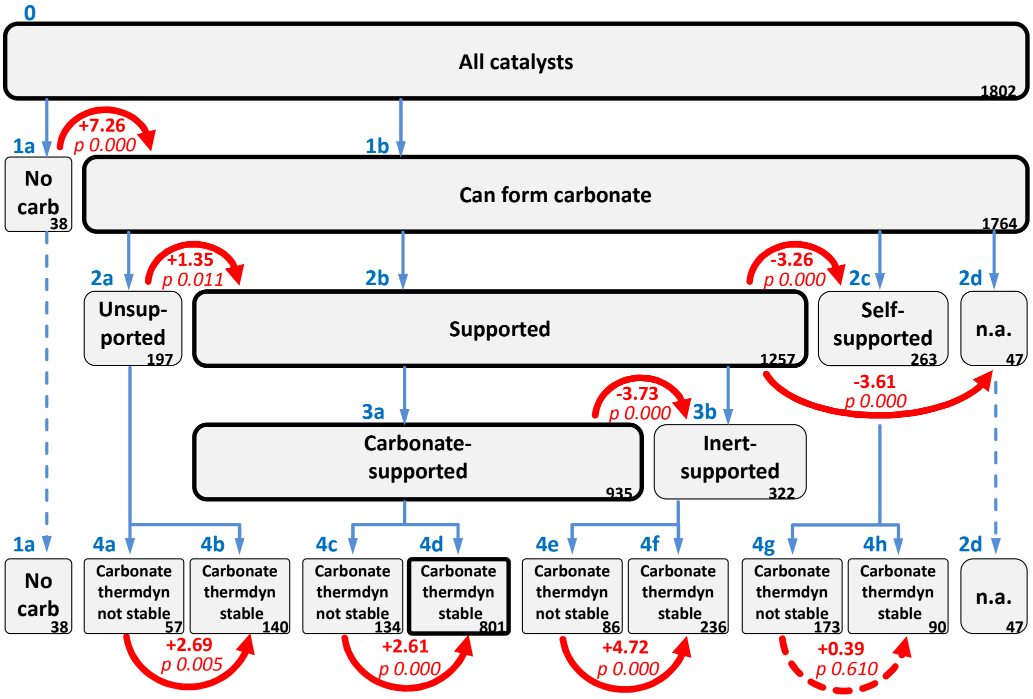

$\bar{Y}_{C2,\ corrected,\ weighted}$	1.56	6.57	8.75	8.72	11.33	4.03	8.35	6.35	6.72	5.86
β_{group} to 4d	+9.75	+4.81	+2.61	+2.61	-	+7.31	+2.95	+4.96	+4.60	+5.44
p-value	*0.000*	*0.000*	*0.000*	*0.000*	-	*0.000*	*0.000*	*0.000*	*0.000*	*0.000*

Supplementary Figure 16 Catalyst property tree with hypotheses results obtained when weighting is applied to compensate for the different observation frequency of different cation combinations. (Note that the weighted mean C_2 yields $\bar{Y}_{C2,corrected,weightes}$ reported in the table were calculated slightly different. First, the yield for each catalyst was compensated for reaction conditions. Then, for each cation combination within a property group, the average yield was calculated. Thereafter, the mean yield over all averaged yields for different cation combinations was computed.)

Supplementary Note 17 Robustness test: Regression towards ln(Y_{C2}) as independent variable

Throughout the statistical analysis, significance of the obtained regression coefficients β_{group} is expressed by the corresponding p-value. The p-value is derived from a t-test, for which normal distribution is assumed. However, the yield distribution of the catalyst property groups deviates from a normal distribution (see yield density plots in Fig. 4 of the main article). The normal distribution can be better fulfilled when the data are transformed using a logarithm.

Therefore, the regression analysis was performed using ln(Y_{C2}) instead of Y_{C2} as the independent variable, according to equation (1).

$$\ln(Y_i) = \beta_0 + \beta_{group} \times D_{group,i} + \beta_T \times \Delta T_i + \beta_{CH4/O2} \times \Delta p_{CH4/O2,i} + U_i \qquad (1)$$

In case that for a catalyst $Y_i = 0.0\%$, the yield was arbitrarily set to $Y = 0.00001\%$, so that the observation can be included when applying the logarithm. The β_{group} regression coefficients relate to differences in ln(Y_{C2}) between property groups and therefore have lower absolute values.

The obtained regression results are presented in Supplementary Figure 17. The results indicate that the main trends of the final model (Fig. 4) are retained. All hypotheses that are statistically significant (p ≤ 0.050) remain significant.

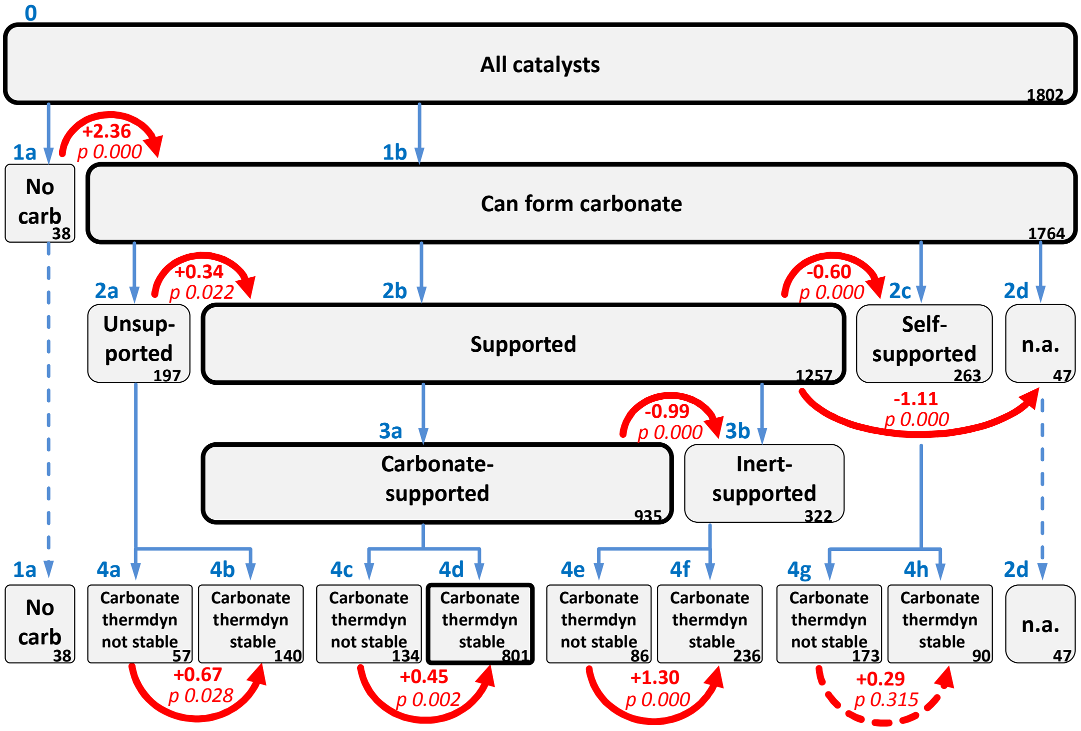

Mean of ln($Y_{C2,uncorr.}$)	-0.61	1.01	1.68	1.92	2.11	0.22	1.47	1.19	1.32	0.71
β_{group} to 4d	+2.90	+1.23	+0.45	+0.45	-	+2.05	+0.71	+1.02	+0.71	+1.41
p-value	*0.000*	*0.000*	*0.001*	*0.002*	-	*0.000*	*0.000*	*0.000*	*0.000*	*0.000*

Supplementary Figure 17 Catalyst property tree with hypotheses results, regression based on ln(Y_{C2}).

Supplementary Note 18 Robustness test: Robust regression

A linear regression that minimizes squared residual is typically affected strongly by outliers. This can be avoided by (partially) excluding outliers from the regression. A so-called robust regression uses realizes the partial exclusion of outliers through M-estimators[28]. The general equations[29] are given by (1) and (2).

$$\hat{\theta}_M = arg \, \underset{\theta}{min} \sum_{i=1}^{n} \delta\left(\frac{r_i(\theta)}{\sigma}\right) \qquad (1)$$

$$\delta(u) = \begin{cases} 1 - [1 - \frac{u^2}{k}]^3 \; for \, \{|u| \leq 4.685\} \\ 1 \; for \, \{|u| > 4.685\} \end{cases} \qquad (2)$$

δ represents a loss function, which handles the input of residuals $r_i(\theta)$ divided by the residuals' standard deviation σ.

Large positive or negative residuals compared to their standard deviation cause large inputs u. If u exceeds k, the largest possible loss functions' output of one is generated. These points are rejected by equation (1) and therefore not considered in the regression.

Regression results using robust regression with compensation for reaction conditions are shown in Supplementary Figure 18. β_{group} coefficients obtained by robust regression show only small deviations compared to the final model (Fig. 4) using ordinary least squared regression. The largest change (0.54 percent points) is obtained for groups 3a/3b. The regression 4a/4b is statistically insignificant using robust regression. However, the main trends retained, showing high robustness of the final model towards implementation of the linear regression.

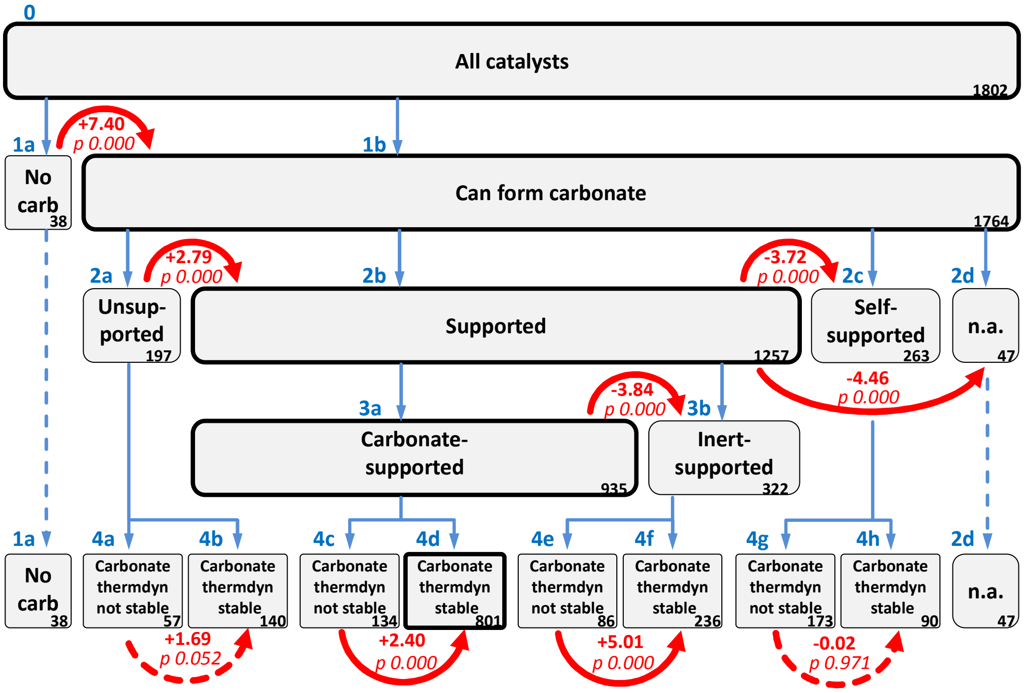

$\bar{Y}_{C2, corrected}$	2.08	6.92	8.52	9.39	11.79	4.41	9.41	6.87	6.96	6.29
β_{group} to 4d	+9.75	+5.01	+3.71	+2.40	-	+7.58	+2.75	+5.00	+4.84	+5.79
p-value	*0.000*	*0.000*	*0.000*	*0.000*	-	*0.000*	*0.000*	*0.000*	*0.000*	*0.000*

Supplementary Figure 18 Catalyst property tree with hypotheses results obtained through robust regression.

Supplementary Note 19 Preparation and testing of Al_2O_3 supported catalysts

Alumina supported catalysts were prepared by wet impregnation of an Al_2O_3 support with aqueous solutions (alkali carbonates, alkaline earth acetates), followed by drying. The procedure was adapted from Kusche et al.[30]

The employed γ-Al_2O_3 support was supplied by Südchemie/Clariant as “Al_2O_3-100” pellets, which were ground and sieved to a size fraction of 200 - 500 µm. Thereafter, the Al_2O_3 was calcined for 12 h at 800 °C in air. The obtained Al_2O_3 had a surface area of 90 m²/g, as evaluated from N_2 physisorption by BET method.

Catalyst loading was chosen as 23.1 wt% of the employed salt in anhydrous state. Only carbonates of alkali elements are sufficiently soluble for aqueous impregnation. Hence, catalysts containing alkaline earth were prepared using acetates. For wet impregnation, typically 1.0 g of Al_2O_3 was stirred in a round flask with 20 ml aqueous salt solution for 5 min. The concentration of the solutions was c = 15 g/l with respect to the respective anhydrous salt. After impregnation, catalysts were dried for 30 min at 60 °C and 60 mbar using a rotary evaporator, followed by a further drying step for 4 h at 150 °C and 37 mbar using a vacuum oven.

The catalysts were tested in a parallel fixed bed reactor setup at Leibniz Institute for Catalysis (LIKAT) Rostock. The setup employs 48 quartz tube reactors with 4 mm inner diameter. The fixed bed is held by a glass wool plug at equal height for each reactor. On the plug the catalyst powder (50 mg) is placed. Above the catalyst powder, pre-calcined SiC granules (300 mg) were placed to ensure the temperature of the feed being equal to heating temperature.

For catalytic testing, the reactors were heated (5 K / min ramp) in OCM feed. Catalytic measurements were performed at holding temperatures in 25 K intervals (450 °C → 5 K / min ramp → 475 °C, …) up to 850 °C. At each holding temperature, catalysts were measured sequentially by analysing the gas phase using a gas chromatograph (Agilent 7890) equipped with PLOT/Q (for CO_2), AL/S (for hydrocarbons) and Molsieve 5 (for H_2, O_2, N_2, and CO) columns.[6] Conversions and selectivities were calculated on feed basis using peak areas of a flame ionisation detector FID (CH_4, C_2H_6, C_2H_4) and a thermal conductivity detector TCD (O_2, N_2, CO_2, CO).

OCM testing was performed in two different feed compositions with similar CH_4 / O_2 ratio. In the “N_2 OCM feed”, N_2 was used as diluent. In the “CO_2 OCM feed”, N_2 was almost completely replaced by CO_2. A low concentration of N_2 was necessary as a reference for calculation. Each feed was tested in independent test runs, starting each time from unused catalysts. The feed compositions are given in Supplementary Table 19. The flow in each reactor channel amounted to 14.7 Nml/min, resulting in a contact time of 0.26 s.

Supplementary Table 19, compositions of OCM feeds employed for catalytic testing of Al_2O_3 supported catalysts

OCM feed	CH_4	O_2	N_2	CO_2
**N_2** OCM feed	26.2 mol%	14.8 mol%	**59.0 mol%**	-
**CO_2** OCM feed	26.2 mol%	14.8 mol%	3.0 mol%	**56.0 mol%**

Supplementary Note 20 Experimental C_2 Yield distributions for typical OCM catalysts Li/MgO and Mn-Na-W/SiO_2

The experimental values contained in the studied dataset feature a broad distribution of reported values even for simple catalyst composition. Supplementary Figure 20 displays the C_2 yield density distributions for two of the most popular catalysts systems, i.e. Li/MgO and variations of the Mn-Na-W/SiO_2 system. The values are shown without any modeling or correction. C_2 yields reported e.g. for Li/MgO range up to ca. 30%, with a mean value of about 12.6%. The broad yield distributions illustrates that a substantial variety in reported yields exists, independent of the definition of the property groups. Thus, this variation is always a major contribution to the yield distribution within any property group.

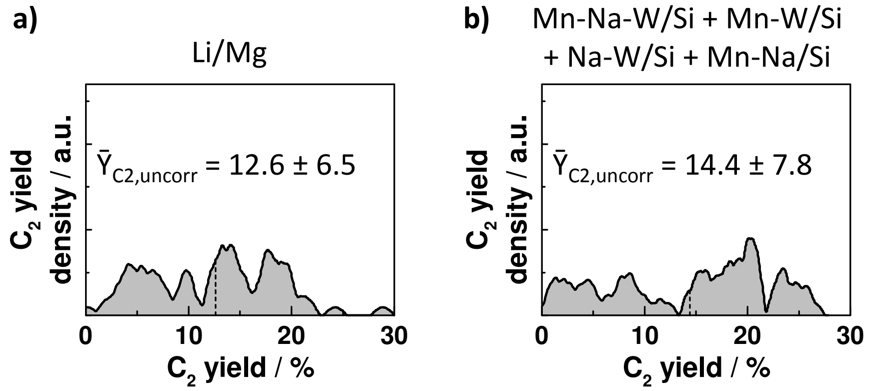

Supplementary Figure 20 C_2 yield-density distribution of the studied literature data as well as respective mean C_2 yield $\bar{Y}_{C2,uncorr}$, and its standard deviation for catalysts with the cation compositions a) Li/Mg and b) variations of Mn-Na-W/Si.

Supplementary References

[1] U. Zavyalova, M. Holena, R. Schlögl, M. Baerns, **2011**, *ChemCatChem*, *3*, 1935.

[2] H. Scheffé, **1999**, *The Analysis of Variance*, John Wiley & Sons Inc.

[3] M. P. Schulze, **2007**, *Beschreibende Statistik*, Oldenbourg Wissenschaftsverlag GmbH, München.

[4] A. J. Onwuegbuzie, L. G. Daniel, **1999**, *Uses and Misuses of the Correlation Coeffcient*, Technical report, College of Education (Valdosta State University, GA).

[5] L. Gordon, **2013**, in *SAS Global Forum Proceedings*, 089-2013

[6] E. V. Kondratenko, M. Schluter, M. Baerns, D. Linke, M. Holena, **2015**, *Catalysis Science & Technology*, *5*, 1668.

[7] M. Santoro, F. A. Gorelli, R. Bini, A. Salamat, G. Garbarino, C. Levelut, O. Cambon, J. Haines, **2014**, *Nature Communications*, *5*, 3761.

[8] G. Tammann, A. Sworykin, **1928**, *Zeitschrift für anorganische und allgemeine Chemie*, *176*, 46.

[9] R. C. Asher, S. J. Gregg, **1960**, *Journal of the Chemical Society (Resumed)*, 5057.

[10] G. F. Hüttig, **1943**, *Kolloid-Zeitschrift*, *104*, 189.

[11] A. Davidson, M. Che, **1992**, *The Journal of Physical Chemistry*, *96*, 9909.

[12] R. Merkle, J. Maier, **2005**, *Zeitschrift für anorganische und allgemeine Chemie*, *631*, 1163.

[13] D. J. M. Bevan, J. P. Shelton, J. S. Anderson, **1948**, *Journal of the Chemical Society (Resumed)*, 1729.

[14] H. Lu, A. Khan, S. E. Pratsinis, P. G. Smirniotis, **2009**, *Energy & Fuels*, *23*, 1093.

[15] R. Schlögl, **2015**, *Angewandte Chemie International Edition*, *54*, 3465.

[16] Y. S. Su, J. Y. Ying, W. H. Green Jr, **2003**, *Journal of Catalysis*, *218*, 321.

[17] P. Schönfeld, **1969**, *Methoden der Ökonometrie: Band I*, Verlag Franz Vehlen GmbH, Berlin.

[18] R. P. Taylor, G. L. Schrader, **1991**, *Industrial & Engineering Chemistry Research*, *30*, 1016.

[19] M. Daneshpayeh, A. Khodadadi, N. Mostoufi, Y. Mortazavi, R. Sotudeh-Gharebagh, A. Talebizadeh, **2009**, *Fuel Processing Technology*, *90*, 403.

[20] N. R. Farooji, A. Vatani, S. Mokhtari, **2010**, *Journal of Natural Gas Chemistry*, *19*, 385.

[21] Z. Taheri, N. Seyed-Matin, A. A. Safekordi, K. Nazari, S. Z. Pashne, **2009**, *Applied Catalysis A: General*, *354*, 143.

[22] R. Ghose, H. T. Hwang, A. Varma, **2014**, *Applied Catalysis A: General*, *472*, 39.

[23] J. D. Hart, **1997**, *Nonparametric Smoothing and Lack-of-Fit Tests*, Springer Science+Business Media, New York.

[24] F. Peracchi, **2011**, *Nonparametric methods*, University of Rome "Tor Vergata" and EIEF, Rome.

[25] T. Ito, J. H. Lunsford, **1985**, *Nature*, *314*, 721.

[26] C. Mirodatos, G. A. Martin, J. C. Bertolini, J. Saint-Just, **1989**, *Catalysis Today*, *4*, 301.

[27] S. Arndt, U. Simon, S. Heitz, A. Berthold, B. Beck, O. Görke, J. D. Epping, T. Otremba, Y. Aksu, E. Irran, G. Laugel, M. Driess, H. Schubert, R. Schomäcker, **2011**, *Topics in Catalysis*, *54*, 1266.

[28] Huber, P. J. et al., **1964**, *The annals of Mathematical Statistics*, 35, 1, 73-101

[29] Verardi, V. and Croux, C., **2008**, Robust regression in Stata, http://dx.doi.org/10.2139/ssrn.1369144

[30] M. Kusche, F. Enzenberger, S. Bajus, et al., *Angewandte Chemie International Edition* **2013**, *52*, 5028.
