# Supplementary material for: A meta-analysis of catalytic literature data reveals property-performance correlations for the OCM reaction
Source: Nat Commun. 2019 Jan 25;10:441. doi: 10.1038/s41467-019-08325-8 (PMC6347636; doi:10.1038/s41467-019-08325-8)
Supplement: Supplementary file 2 — Description of Additional Supplementary Files [file 41467_2019_8325_MOESM2_ESM.pdf]

Description of Additional Supplementary Files

File Name: Supplementary Data 1
Description: Corrected OCM dataset employed in the current study.

File Name: Supplementary Data 2
Description: Element property table with basic physical and chemical properties of chemical elements and their compounds as assembled from literature and own measurements.

File Name: Supplementary Data 3
Description: Table that lists for each catalyst compound in the OCM dataset the assigned property descriptors.

File Name: Supplementary Data 4
Description: Table that lists for each catalyst in the OCM dataset the assigned property groups that were obtained via application of the sorting rules.

File Name: Supplementary Data 5
Description: Table that provides for the final model a list of catalyst compositions (i.e. cation combinations) contained in each property group.
